# Anti-Inflammatory Polyoxygenated Steroids from the Soft Coral *Lobophytum michaelae*

**DOI:** 10.3390/md16030093

**Published:** 2018-03-13

**Authors:** Chiung-Yao Huang, Wan-Ru Tseng, Atallah F. Ahmed, Pei-Lun Chiang, Chi-Jen Tai, Tsong-Long Hwang, Chang-Feng Dai, Jyh-Horng Sheu

**Affiliations:** 1 Department of Marine Biotechnology and Resources, National Sun Yat-sen University, Kaohsiung 804, Taiwan; huangcy@mail.nsysu.edu.tw (C.-Y.H.); m025020010@student.nsysu.edu.tw (W.-R.T.); 2 Department of Pharmacognosy, College of Pharmacy, King Saud University, Riyadh 11451, Saudi Arabia; afahmed@ksu.edu.sa; 3 Department of Biochemistry, University of Toronto, Toronto, ON M5S 1A8, Canada; sharonjiang1996@hotmail.com; 4 Doctoral Degree Program in Marine Biotechnology, National Sun Yat-sen University and Academia Sinica, Kaohsiung 804, Taiwan; jean801023@hotmail.com; 5 Graduate Institute of Natural Products, College of Medicine, Chang Gung University, Taoyuan 333, Taiwan; htl@mail.cgu.edu.tw; 6 Research Center for Chinese Herbal Medicine, Research Center for Food and Cosmetic Safety, and Graduate Institute of Health Industry Technology, College of Human Ecology, Chang Gung University of Science and Technology, Taoyuan 333, Taiwan; 7 Department of Anesthesiology, Chang Gung Memorial Hospital, Taoyuan 333, Taiwan; 8 Institute of Oceanography, National Taiwan University, Taipei 112, Taiwan; corallab@ntu.edu.tw; 9 Graduate Institute of Natural Products, Kaohsiung Medical University, Kaohsiung 807, Taiwan; 10 Department of Medical Research, China Medical University Hospital, China Medical University, Taichung 404, Taiwan; 11 Frontier Center for Ocean Science and Technology, National Sun Yat-sen University, Kaohsiung 804, Taiwan

**Keywords:** soft coral, *Lobophytum michaelae*, cytotoxicity, anti-inflammatory activity

## Abstract

Three new polyoxygenated steroids, michosterols A–C (**1**–**3**), and four known compounds (**4**–**7**) were isolated from the ethyl acetate (EtOAc) extract of the soft coral *Lobophytum michaelae*, collected off the coast of Taitung. The structures of the new compounds were elucidated on the basis of spectroscopic analyses and comparison of the nuclear magnetic resonance (NMR) data with related steroids. The cytotoxicity of compounds **1**–**3** against the proliferation of a limited panel of cancer cell lines was assayed. Compound **1** was found to display moderate cytotoxicity against adenocarcinomic human alveolar basal epithelial (A549) cancer cells. It also exhibited potent anti-inflammatory activity by suppressing superoxide anion generation and elastase release in *N*-formyl-methionyl-leucyl-phenylalanine/cytochalasin B (fMLP/CB)-stimulated human neutrophils. Furthermore, **3** could effectively inhibit elastase release, as well.

## 1. Introduction

Previous chemical investigations on the octocorals of the genus *Lobophytum* have led to the isolation of structurally unique steroids [1,2,3,4,5,6], some of which have been shown to exhibit cytotoxic [2,3,5,6] and anti-inflammatory [2,5] activities. For the purpose of searching for bioactive compounds, we have previously investigated the chemical constituents of soft corals of the genus *Lobophytum* growing in Taiwanese waters, which resulted in the discovery of a series of bioactive natural products [6,7,8,9,10,11]. Previous investigations also have shown that the soft coral *Lobophytum michaelae* could produce bioactive cembranolides with cytotoxicity towards cancer cell lines [12,13,14]. With the aim of discovering more bioactive marine natural products for new drug development in the future, we again investigated the chemical constituents of a Formosan soft coral *L. michaelae*. From this study, we have isolated three new polyoxygenated steroids, michosterols A–C (**1**–**3**), together with four known compounds: brassicasterol (**4**) [15], 24*S*-methylcholesterol (**5**) [16], 23-demethylgorgosterol (23-demethylgorgost-5-en-3β-ol) (**6**) [17,18,19] and gorgosterol (**7**) [20]. Extensive spectroscopic analyses, including mass spectrometry, 1D and 2D NMR spectroscopy (Supplementary Materials, Figures S1–S26), and comparison of spectroscopic data of the new compounds with those of the previously reported structurally-related compounds have allowed us to establish the structures of **1**–**3**. Compound **1** possesses a double bond between C-16 and C-17 and an unusual side-chain with a hydroperoxyl group at C-20, whereas **2** has this group at C-16 and an uncommon olefinic structure at C-17 and C-20. The in vitro cytotoxicity of **1**–**3** against three cancer cell lines, adenocarcinomic human alveolar basal epithelial (A549), human colorectal adenocarcinoma (DLD-1) and human prostatic carcinoma (LNCap), was evaluated. The abilities of compounds **1**–**3** to inhibit superoxide anion generation and elastase release in *N*-formyl-methionyl-leucyl-phenylalanine/cytochalasin B (fMLP/CB)-stimulated human neutrophils was also assayed.

## 2. Results and Discussion

**Figure 1:**
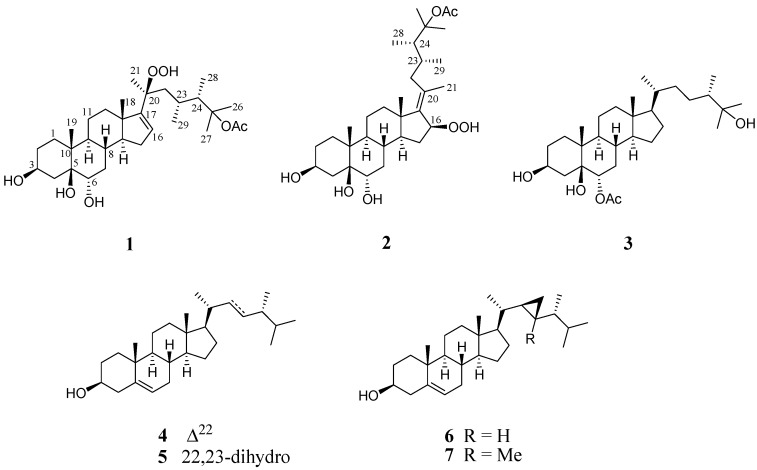
Structures of compounds **1**–**7**.

The frozen bodies of *L. michaelae* were sliced and exhaustively extracted with ethyl acetate (EtOAc). The EtOAc extract was separated by repeated gravity column chromatography and high-performance liquid chromatography (HPLC) to afford three new and four known triterpenoids steroids **1**–**7** (Figure 1). 

**Figure 2:**
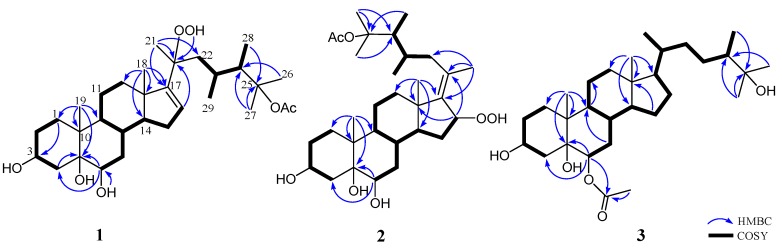
Selected COSY and HMBC correlations of **1**–**3**.

The molecular formula of **1**, an amorphous solid, was determined as C_31_H_52_O_7_ based on the [M + Na]^+^ ion peak obtained by high-resolution electron spray ionisation mass spectrometry (HRESIMS), implying six degrees of unsaturation. The ^13^C NMR spectrum showed 31 carbon signals, including an ester carbonyl (δ_C_ 171.9, C), a double bond (δ_C_ 157.6, C and 127.0, CH), two oxymethines (δ_C_ 71.8 and 67.7, each CH) and three oxygenated *sp*^3^ quaternary carbons (δ_C_ 87.2, 85.6, and 78.1) (Table 1). The ^1^H NMR spectrum in conjunction with the heteronuclear single quantum coherence (HSQC) spectrum revealed the presence of eight methyl groups (δ_H_ 2.03 (3H, s), 1.50 (3H, s), 1.41 (3H, s), 1.31 (3H, s), 0.96 (6H, s), 0.93 (3H, d, *J* = 6.8 Hz) and 0.88 (3H, d, *J* = 7.2 Hz)), an olefinic methine proton (δ_H_ 5.70 (1H, d, *J* = 2.0 Hz)) and a hydroperoxy group signal at δ_H_ 8.06 (br s). Thus, the remaining four unsaturations of **1** corresponded to a tetracyclic skeleton. In the correlation spectroscopy (COSY) spectrum, it was possible to identify three different structural units extending from C-1 to C-4; C-6 to both C-12 and C-16 through C-8; and C-22 to both C-28 and C-29 through C-23 (Figure 2). From the heteronuclear multiple-bond correlation (HMBC) spectrum, the correlations of H_3_-19 to C-1, C-5, C-9 and C-10, H_3_-18 to C-12, C-13, C-14 and C-17, H-6 to C-4 and C-5, H-16 to C-20, H_3_-21 to C-17, C-20 and C-22, both H_3_-26 and H_3_-27 to C-24 and H_3_-28 to C-25 permitted the establishment of the carbon skeleton of a 23,24-dimethycholestane (Figure 2). The hydroperoxy group positioned at C-20 was confirmed from the HMBC correlation of the hydroperoxy proton δ_H_ 8.06 (br s) to the oxygenated carbon at δ_C_ 85.6; hence, the acetoxy group was positioned at C-25 (δ_C_ 87.2). The planar structure of **1** was thus established unambiguously.

**Figure 3:**
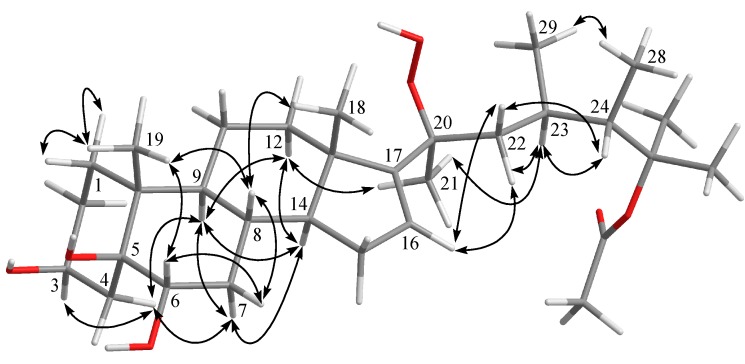
Selected NOESY correlations of compound **1**.

**Figure 4:**
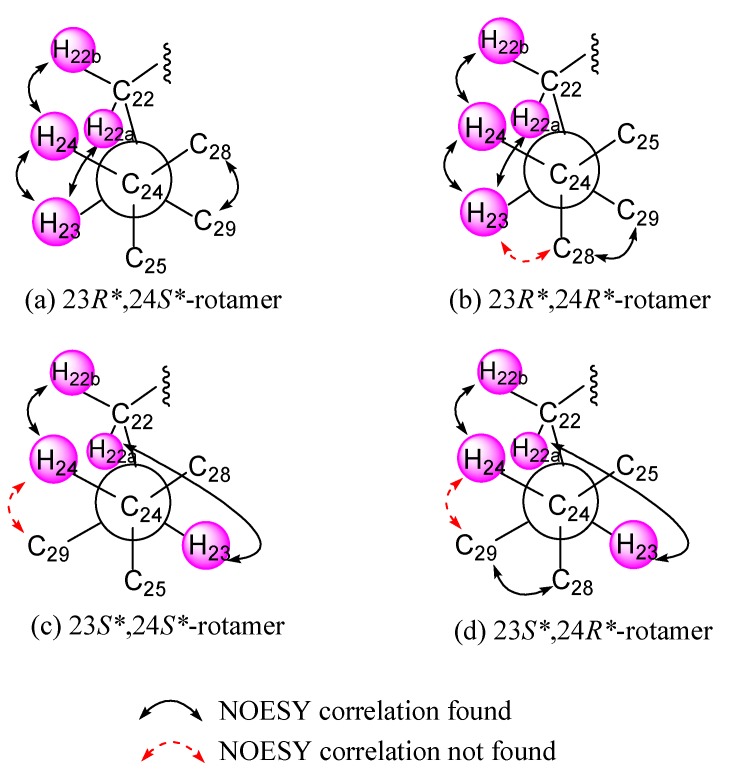
(**a**–**d**) C-24/C-25 rotamers of compound **1**.

The relative configuration of **1** was deduced by interpretation of the nuclear Overhauser effect (NOE) correlations (Figure 3), analysis of ^3^*J*_H-H_ values and comparison of carbon chemical shifts. As depicted in Figure 3, it was found that the NOE interactions displayed by both H_3_-18 and H_3_-19 with H-8 and H_3_-19 with H-6, while one of the methylene protons at C-7 (δ_H_ 1.85, m), showed NOESY correlations with both of H-6 and H-8. Therefore, assuming the β-orientation of H_3_-19, H-6, H-7 at δ_H_ 1.85, H-8 and H_3_-18 should be positioned on the β-face, while the other H-7 (δ_H_ 1.09, q, *J* = 12.0 Hz) was assigned as H-7α. Moreover, H-14 showed NOESY correlations with H-7α, H-9 and one proton of H_2_-12 (δ_H_ 2.06, m); whereas the latter proton was NOE correlated with H_3_-21. This reflects theα-orientations of H-9, H-14 and H_3_-21 and, consequently, the β-orientation of the hydroperoxy group at C-20 of the side chain. Further, H_3_-21 exhibited NOESY correlation with H-23; and H-23 expressed NOE interaction with H-24 as did H_3_-28 with H_3_-29, respectively, while no NOE interaction was found for H-23 with H_3_-28 and for H-24 with H_3_-29. Thus, the 23R*, 24S* relative configurations were revealed (Figure 3) and further supported by the comparison of the NOE interactions in **1** with those anticipated in its other three 23,24-rotamers (Figure 4). Finally, the configurations of C-3, C-5 andC-6 were elucidated by comparison of the ^1^H NMR chemical shifts and coupling constants of H-3 and H-6 with those of related steroids (Table 2). The δ and *J* values of H-3 (δ_H_ 4.24, s) and H-6 (δ_H_ 3.74–3.81, dd, *J* = 12.0, 4.8 Hz) of known compound 5β-cholestane-3β,5,6α-triol [21,22] were found to be similar to the corresponding H-3 (δ_H_ 4.27, br s) and H-6 (δ_H_ 3.82, dd, *J* = 12.0, 4.8 Hz) of **1** (Table 2). Consequently, the relative configuration of **1** was determined unequivocally. Since it has been known for quite a long time that both H_3_-18 and H_3_-19 should be positioned on the β-face for natural steroids, thus the absolute configuration of **1** should be the same as shown in Structure **1**.

**Figure 5:**
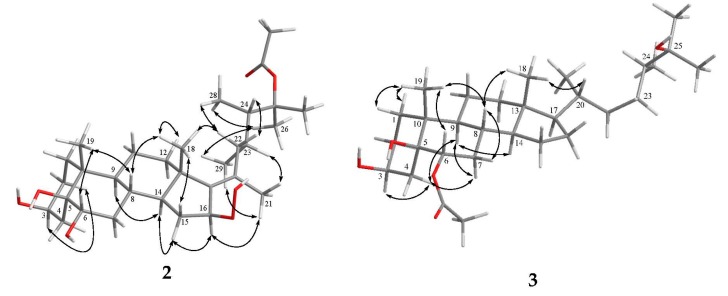
Selected NOESY correlations of compounds **2** and **3**.

Analysis of the ^13^C NMR and HRESIMS spectral data of **2** revealed that it has the same molecular formula as that of **1**. The ^1^H and ^13^C NMR spectra of **2** are similar to those of **1**, and the molecular skeleton of **2** was further established by HMBC correlations from H_3_-21 to C-17 (δ_C_ 143.6, C), C-20 (δ_C_ 132.8, C) and C-22 (δ_C_ 41.2, CH_2_); H-16 (δ_H_ 4.96, dd, *J* = 7.5, 7.5 Hz) to C-13 (δ_C_ 44.1, C) and C-20; and the COSY correlation between H_2_-15 and H-16 (Figure 2). The above results showed that an olefinic proton signal (δ_H_ 5.70, d, *J* = 2.0 Hz) in **1** was replaced by an oxymethine proton (δ_H_ 4.96, dd, *J* = 7.5, 7.5 Hz), which also showed correlation with the signal of C-16 (δ_C_ 86.1) in the HSQC spectrum of **2**. Further, a proton signal appearing at δ_H_ 8.91 (br s) was found not to be correlated with any carbon signal in the HSQC spectrum. Thus, this should be the signal of a hydroperoxy group substituted at C-16, as confirmed by the downfield shift of this carbon in **2** in comparison with the chemical shifts of the corresponding carbons of two 16-hydroxy analogues, δ_C_ 72.3 for faccisteroid B [23] and 71.8 for hippuristerone L [24]. The comparison of the ^1^H NMR chemical shifts and the analysis of NOESY correlations of **2** revealed the same configurations at C-3, C-5, C-6, C-8, C-9, C-10, C-13, C-14, C-23 and C-24 as those of **1**. Further, NOESY correlations from H_3_-18 to H-8, H-15β (δ_H_ 1.69, m) and H-22β (δ_H_ 1.76 m) were observed, while H-15α (δ_H_ 2.08 m) was correlated with H-14 and H-16 and H-16 with H_3_-21, suggesting a β-orientation of hydroperoxy group at C-16 and the *E* geometry of Δ^17(20)^ in **2**. On the basis of the above findings and the careful analysis of NOESY correlations (Figure 5), the structure of **2** was determined as illustrated in Figure 1.

Michosterol C (**3**) was isolated as a white powder and has a molecular formula of C_30_H_52_O_5_ by the analysis of HRESIMS. The IR spectrum of **3** also revealed the presence of hydroxy (3416 cm^−1^) and ester carbonyl (1718 cm^−1^) groups. Comparison of the ^1^H and ^13^C NMR data (Table 1) of compounds **2** and **3** pointed out that the A–C rings of **3** were similar to those of **2**, with the exception of signals assigned to C-6, where the hydroxymethine (δ_H_ 3.80, dd, *J* = 12.0, 5.0 Hz; δ_C_ 71.7) in **2** was replaced by an acetoxymethine (δ_H_ 4.97, dd, *J* = 12.0, 4.8 Hz; δ_C_ 75.8) in **3**. The acetoxy group substitution at C-6 was determined by the HMBC correlations of H-6 (δ_H_ 4.97) with C-4 (δ_C_ 31.1, CH_2_), C-5 (δ_C_ 77.4, C) and an acetoxy carbonyl carbon (δ_C_ 171.9, C), and further confirmed by the COSY spectrum (Figure 2). Furthermore, the signal of a hydroperoxy-bearing methine group at C-16 (δ_H_ 4.96, dd, *J* = 7.5, 7.5 Hz; δ_C_ 86.1) in **2** was replaced by signals of a methylene group (H_2_-16:δ_H_ 1.28 m and 1.84 m; δ_C_ 28.0). Comparison of the NMR data of **3**, measured in CD_3_OD (see the Experimental Section) with those of (24S)-ergostane-6-acetate-3β,5α,6β,25-tetraol [21] indicated that the planar structure and the configurations of the side chain of both compounds are the same. Furthermore, the configurations of C-3, C-5 and C-6 were elucidated by comparison of the ^1^H NMR coupling constants with the related steroids (Table 1 and Table 2). The *J* values of H-3 (δ_H_ 4.11, br s) and H-6 (δ_H_ 4.97, dd, *J* = 12.0, 4.8 Hz) of compound **3** were identical to the corresponding H-3 (δ_H_ 4.27, br s) and H-6 (δ_H_ 3.82, dd, *J* = 12.0, 4.8 Hz) of **1**. In addition, NOESY correlations of H_3_-19 with both H-6 and H-8, H-8 with H-7β (δ_H_ 1.81, m), H-7α (δ_H_ 1.07, m) with H-4α (δ_H_ 1.92, m) and H-9 and H-4α with H-3 confirmed the α-orientations of the H-3 and 6-OAc group and the β-orientation of the 5-OH group (Figure 5). Thus, the structure of **3** was established as shown in Figure 1.

The cytotoxicity of the isolates **1**–**3** against A549, DLD-1 and LNCap cancer cells was assayed. The results showed that only compound **1** exhibited a moderate cytotoxicity effect against the A549 cell line with an IC_50_ value of 14.9 ± 5.7 μg/mL. The other compounds were found not to be cytotoxic against the above cancer cell lines (IC_50’_s > 20 μg/mL).

The anti-inflammatory activities of the new compounds **1**–**3** on pro-inflammatory responses were evaluated by measuring their ability to suppress fMLP/CB-induced superoxide anion (O_2_^−•^) generation and elastase release in human neutrophils. From the results (Table 3), compound **1** showed significant inhibitory effects against superoxide anion generation and elastase release in fMLP/CB-stimulated cells at the 10 μM concentration tested, with the IC_50_ values being 7.1 ± 0.3 μM and 4.5 ± 0.9 μM, respectively. Further, compound **3** exhibited stronger inhibitory activities against elastase release, with the IC_50_ values of 0.9 ± 0.1 μM.

## 3. Experimental Section

### 3.1. General Experimental Procedures

The specific optical rotation values and IR spectral absorptions were recorded on a JASCO P-1020 digital polarimeter (JASCO Corporation, Tokyo, Japan) and a JASCO J-815 spectrophotometer (JASCO Corporation), respectively. A Varian 400MR FT-NMR or Varian Unity INOVA-500 FT-NMR (Varian Inc., Palo Alto, CA, USA) was applied to record the ^1^H and ^13^C NMR spectra with the chemical shifts shown as ppm referenced to the solvent residue of CDCl_3_ (δ_H_ 7.26 ppm and δ_C_ 77.0 ppm) and CD_3_OD (δ_H_ 3.31 ppm and δ_C_ 49.0 ppm), respectively. A Bruker APEX II mass spectrometer equipped with an ESI ionization source (Bruker, Bremen, Germany) was used for acquiring high-resolution mass data. The HPLC system used in this study was composed of a Hitachi L-2455 instrument (Hitachi Ltd., Tokyo, Japan) equipped with a reversed-phase (RP-18) column (ODS-3, 5 μm, 250 × 20 mm, Sciences Inc., Tokyo, Japan).

### 3.2. Animal Material 

The collection of the soft coral *Lobophytum michaelae* Tixier-Durivault (1956) was performed off the coast of Jihui Fishing Port, Taitung County, Taiwan, in March 2013. The organism was stored in a freezer at −20 °C until extraction. Prof. C.-F. Dai performed the species identification. A voucher specimen (JiH-201314) of this soft coral has been deposited at National Sun Yat-sen University.

### 3.3. Extraction and Isolation

The frozen bodies of *L. michaelae* (3.2 kg, wet wt) were sliced and exhaustively extracted with EtOAc (3 × 4 L). The EtOAc extract (5.95 g) was chromatographed over silica gel by column chromatography and eluting with EtOAc in *n*-hexane (0–100%, stepwise), followed by acetone in EtOAc (50–100%, stepwise) to yield 22 fractions. Fraction 9 was eluted with acetone/*n*-hexane (1:6) on silica gel to give twelve subfractions (SFr.9-1–SFr.9-12), and SFr.9-3 was separated on RP-18 gel, using MeOH/H_2_O (7:1) to give ten subfractions (SFr.9-3-1–SFr.9-3-10); SFr.9-3-2 was subjected to PR-HPLC with CH_3_CN/MeOH (1:10) to obtain compounds **4** (1.9 mg), **5** (3.6 mg), **6** (2.9 mg) and **7** (3.4 mg). Fraction 14, eluted with *n*-hexane/EtOAc (1:4), also was rechromatographed over a Sephadex LH-20 column, using acetone as the mobile phase. In turn, SFr. 14-2 was separated on silica gel, using acetone/*n*-hexane (1:4) to give two subfractions SFr. 14-2-1–SFr.14-2-2. SFr.14-2-2 was isolated on RP-HPLC using MeOH/H_2_O (10:1) to give **3** (32.3 mg). SFr.14-4 was further purified by reversed phase HPLC using methanol/H_2_O (4: 1) to obtain **1** (5.6 mg) and **2** (1.6 mg).

Michosterol A (**1**): amorphous solid; [α]${}_{D}^{25}$ −5 (*c* 1.25, CHCl_3_); IR (neat) *v*_max_ 3393, 2972, 2938, 1712, 1453, 1371, 1270, 1153, 1051 and 757 cm^–1^; ^13^C and ^1^H NMR data (400 MHz; CDCl_3_), see Table 1; ESIMS *m*/*z* 559 [M + Na]^+^; HRESIMS *m*/*z* 559.3601 [M + Na]^+^ (calcd. for C_31_H_52_O_7_Na, 559.3605).

Michosterol B (**2**): amorphous solid; [α]${}_{D}^{25}$ −51 (*c* 0.25, CHCl_3_); IR (neat) *v*_max_ 3418, 2933, 1713, 1455, 1375, 1259, 1053 and 756 cm^–1^; ^13^C and ^1^H NMR data (500 MHz; CDCl_3_), see Table 1; ESIMS *m*/*z* 559 [M + Na]^+^; HRESIMS *m*/*z* 559.3603 [M + Na]^+^ (calcd. for C_31_H_52_O_7_Na, 559.3605).

Michosterol C (**3**): white powder; [α]${}_{D}^{25}$ +34 (*c* 0.91, CHCl_3_); IR (neat) *v*_max_ 3416, 2942, 2870, 1718, 1454, 1375, 1260, 1049 and 756 cm^–1^; ^13^C and ^1^H NMR data (400 MHz; CDCl_3_), see Table 1; ^1^H NMR (CD_3_OD, 400 MHz): δ_H_ 4.94 (1H, dd, *J* = 12.0, 5.2 Hz, H-6), δ_H_ 4.14 (1H, br s, H-3), δ_H_ 2.07 (3H, s, H-6-OAc), δ_H_ 2.04 (1H, m, H-12a), δ_H_ 1.97 (2H, m, H_2_-4), δ_H_ 1.89 (1H, m, H-16a), δ_H_ 1.84 (1H, m, H-7a), δ_H_ 1.83 (1H, m, H-1a), δ_H_ 1.76 (1H, m, H-23a), δ_H_ 1.65 (1H, m, H-2a), δ_H_ 1.59 (2H, m, H-2b and H-8), δ_H_ 1.58 (1H, m, H-15a), δ_H_ 1.56 (1H, m, H-22a), δ_H_ 1.48 (1H, m, H-11a), δ_H_ 1.40 (1H, m, H-20), δ_H_ 1.36 (2H, m, H-1b and H-11b), δ_H_ 1.32 (1H, s, H-24), δ_H_ 1.31 (1H, m, H-16), δ_H_ 1.20 (1H, m, H-12b), δ_H_ 1.19 (1H, m, H-17), δ_H_ 1.14 (1H, m, H-14), δ_H_ 1.13 (3H, s, H_3_-27), δ_H_ 1.12 (1H, m, H-15), δ_H_ 1.11 (3H, s, H_3_-26), δ_H_ 1.07 (1H, m, H-7b), δ_H_ 0.97 (3H, s, H_3_-19), δ_H_ 0.96 (3H, d, *J* = 5.2 Hz, H_3_-21), δ_H_ 0.95 (1H, m, H-22b), δ_H_ 0.89 (3H, d, *J* = 6.8 Hz, H_3_-28), δ_H_ 0.78 (1H, m, H-23), δ_H_ 0.71 (3H, s, H_3_-18); ^13^C NMR (CD_3_OD, 100 MHz): 173.0 (C, C-6-OAc), 78.1 (C, C-5), 76.3 (CH, C-6), 74.4 (C, C-25), 68.2 (CH, C-3), 57.7 (CH, C-14), 57.6 (CH, C-17), 46.6 (CH, C-24), 44.4 (CH, C-9), 44.1 (C, C-13), 43.2 (C, C-10), 41.4 (CH_2_, C-12), 38.0 (CH, C-20), 36.5 (CH_2_, C-22), 35.3 (CH, C-8), 34.8 (CH_2_, C-7), 32.1 (CH_2_, C-4), 29.5 (CH_2_, C-16), 29.4 (CH_2_, C-23), 28.5 (CH_2_, C-2), 27.4 (CH_3_, C-27), 26.7 (CH_2_, C-1), 26.0 (CH_3_, C-26), 25.5 (CH_2_, C-15), 23.0 (CH_2_, C-11), 21.6 (CH_3_, C-6-OAc), 19.8 (CH_3_, C-21), 17.9 (CH_3_, C-19), 15.5 (CH_3_, C-28), 12.8 (CH_3_, C-18); ESIMS *m*/*z* 515 [M + Na]^+^; HRESIMS *m*/*z* 515.3708 [M + Na]^+^ (calcd. for C_30_H_52_O_5_Na, 515.3707).

### 3.4. Cytotoxicity Assay

A549, DLD-1 and LNCap cancer cells were obtained from the American Type Culture Collection (ATCC; Manassas, VA, USA). Evaluation of cytotoxicity for the isolated metabolites from *L. michaelae* was performed according to the Alamar Blue assay [25,26]. 

### 3.5. Human Neutrophil Superoxide Anion Generation and Elastase Release

Human neutrophils were isolated through dextran sedimentation and Ficoll centrifugation. By following the procedures described previously, the assay of superoxide anion generation was measured from the SOD-inhibitable reduction of ferricytochrome C. The elastase release experiment was performed according to methoxy-succinyl-alanyl-alanyl-prolyl-valine-*p*-nitroanilide (MeO-Suc-Ala-Ala-Pro-Val-*p*-nitroanilide) as the enzyme substrate. Results are expressed as the mean ± SEM, and comparisons were made using Student’s *t*-test [27,28].

## 4. Conclusions

Our investigation demonstrated that a Taiwanese soft coral *L.* *michaelae* could be a good source of bioactive substances. Three new polyoxygenated steroids, michosterols A–C (**1**–**3**), were isolated. Compound **1** has an unusual side-chain, and **2** possesses a 17,20-double bound with rare substituents at both sp^2^ carbons. Compound **1** exhibited moderate anti-inflammatory activities in the inhibition of superoxide anion generation and elastase release in the fMLP/CB-stimulated human neutrophils, and **3** exhibited significant inhibitions toward elastase release, as well. Thus, compounds **1** and **3** can be considered as promising leads in the development of anti-inflammatory drugs.

## Figures and Tables

**Table 1:** ^13^C and ^1^H NMR data of compounds **1**–**3** in CDCl_3_.

	1		2		3	
Position	δ_C_ *^a^*, Mult. *^b^*	δ_H_ *^c^*, Mult. (*J*) *^d^*	δ_C_ *^e^*, Mult.	δ_H_ *^f^*, Mult. (*J*)	δ_C_ *^a^*, Mult.	δ_H_ *^c^*, Mult. (*J*)
1	25.2, CH_2_	1.35 m; 1.81 m	25.1, CH_2_	1.33 m; 1.81 m	25.1, CH_2_	1.34 m; 1.82 m
2	27.7, CH_2_	1.59 m; 1.65 m	27.8, CH_2_	1.59 m	27.6, CH_2_	1.59 m
3	67.7, CH	4.27 br s	67.7, CH	4.25 br s	66.9, CH	4.11 br s
4	30.0, CH_2_	α: 1.93 m; β: 1.87 m	29.9, CH_2_	1.81 m; 1.92 m	31.1, CH_2_	1.92 br s
5	78.1, C		77.8, C		77.4, C	
6	71.8, CH	3.82 dd (12.0, 4.8)	71.7, CH	3.80 dd (12.0, 5.0)	75.8, CH	4.97 dd (12.0, 4.8)
7	34.5, CH_2_	α: 1.09 q (12.0) β: 1.85 m	34.6, CH_2_	1.03 m; 1.88 m	33.6, CH_2_	1.07 m 1.81 m
8	32.4, CH	1.76 m	33.0, CH	1.63 m	33.6, CH	1.56 m
9	43.3, CH	1.34 m	42.9, CH	1.27 m	42.7, CH	1.25 m
10	41.1, C		40.9, C		41.6, C	
11	21.5, CH_2_	1.36 m; 1.51 m	22.0, CH_2_	1.38 m; 1.52 m	21.4, CH_2_	1.29 m; 1.42 m
12	35.8, CH_2_	α: 2.06 m; β: 1.66 m	38.7, CH_2_	1.44 m; 2.27 m	39.7, CH_2_	1.13 m; 1.99 br d (12.8)
13	47.4, C		44.1, C		42.6, C	
14	57.8, CH	1.52 m	51.7, CH	1.09 m	56.2, CH	1.07 m
15	31.0, CH_2_	1.90 m; 2.12 m	29.4, CH_2_	1.69 m; 2.08 m	24.0, CH_2_	1.05 m; 1.53 m
16	127.0, CH	5.70 d (2.0)	86.1, CH	4.96 dd (7.5, 7.5)	28.0, CH_2_	1.28 m; 1.84 m
17	157.6, C		143.6, C		55.8, CH	1.13 m
18	17.9, CH_3_	0.96 s	17.7, CH_3_	0.99 s	11.9, CH_3_	0.64 s
19	17.2, CH_3_	0.96 s	17.0, CH_3_	0.94 s	17.0, CH_3_	0.97 s
20	85.6, C		132.8, C		36.2, CH	1.36 m
21	22.4, CH_3_	1.31 s	19.4, CH_3_	1.71 s	18.9, CH_3_	0.92 d (6.4)
22	44.5, CH_2_	α: 1.99 m β: 1.42 m	41.2, CH_2_	1.76 m; 2.43 dd (10.0, 13.5)	34.8, CH_2_	0.92 m; 1.50 m
23	26.3, CH	2.05 m	29.6, CH	2.10 m	27.8, CH_2_	0.77 m; 1.68 m
24	46.1, CH	2.28 m	40.7, CH	2.24 m	45.1, CH	1.27 m
25	87.2, C		87.0, C		73.6, C	
26	24.1, CH_3_	1.41 s	24.0, CH_3_	1.41 s	26.0, CH_3_	1.14 s
27	25.8, CH_3_	1.50 s	25.3, CH_3_	1.43 s	27.2, CH_3_	1.15 s
28	9.3, CH_3_	0.88 d (7.2)	9.3, CH_3_	0.81 d (7.0)	14.8, CH_3_	0.88 d (6.8)
29	18.0, CH_3_	0.93 d (6.8)	16.7, CH_3_	0.86 d (7.0)		
OAc	22.7, CH_3_	2.03 s	22.7, CH_3_	2.00 s	21.3, CH_3_	2.03 s
	171.9, C		171.2, C		171.9, C	
6-OH		4.11 br s		4.01 br s		
20-OOH		8.06 br s		8.91 br s		

*^a^* Spectrum recorded at 100 MHz; *^b^* attached protons were deduced by the DEPT experiment; *^c^* spectrum recorded at 400 MHz; *^d^* *J* values (in Hz) in parentheses; *^e^* spectrum recorded at 125 MHz; *^f^* spectrum recorded at 500 MHz.

**Table 2:** The ^1^H NMR chemical shifts and coupling constants of the H-3 and H-6 of compound **1** and the related steroids [22].

	Michosterol A (1)	5α-Cholestane-3β,5,6α-Triol	5α-Cholestane-3β,5,6β-Triol	5β-Cholestane-3β,5,6α-Triol	5β-Cholestane-3β,5,6β-Triol
H-3	4.27, br s	4.06, m	4.09, m	4.24, s	4.14, s
H-6	3.82, dd, *J* = 12.0, 4.8 Hz	3.64, dd	3.53, s	3.74–3.81, dd, *J* = 12.0, 4.8 Hz	3.56, m

**Table 3:** Inhibitory effects of compounds **1**–**3** on superoxide anion generation and elastase release in *N*-formyl-methionyl-leucyl-phenylalanine/cytochalasin B (fMLP/CB)-induced human neutrophils at 10 μM.

Compound	Superoxide Anion Generation			Elastase Release		
	IC_50_ (μM) *^a^*	Inh% *^b^*		IC_50_ (μM) *^a^*	Inh% *^b^*	
1	7.1 ± 0.3	63.1 ± 1.1	***	4.5 ± 0.9	91.7 ± 3.1	***
2	>10	14.7 ± 5.7		>10	31.8 ± 5.0	**
3	>10	17.8 ± 2.8	**	0.9 ± 0.1	95.4 ± 3.6	***
Idelalisib	0.07 ± 0.01	102.8 ± 2.2	***	0.3 ± 0.1	99.6 ± 4.2	***

***^a^*** Concentration necessary for 50% inhibition (IC_50_); *^b^* percentage of inhibition (Inh%) at a 10 μM concentration. Results are presented as the mean ± S.E.M. (*n* = 3–4). ** *p* < 0.01, *** *p* < 0.001 compared with the control value.

## Supplementary Materials

HRESIMS, ^1^H, ^13^C, HSQC, COSY, HMBC and NOESY NMR spectra of new compounds **1**–**3** are available online at http://www.mdpi.com/1660-3397/16/3/93/s1. Figure S1: HRESIMS spectrum of **1**, Figure S2: ^1^H NMR spectrum of **1** in CDCl_3_ at 400 MHz, Figure S3: ^13^C NMR spectrum of **1** in CDCl_3_ at 100 MHz, Figure S4: HSQC NMR spectrum of **1** in CDCl_3_ at 400 MHz, Figure S5: COSY NMR spectrum of **1** in CDCl_3_ at 400 MHz, Figure S6: HMBC NMR spectrum of **1** in CDCl_3_ at 400 MHz, Figure S7: NOESY NMR spectrum of **1** in CDCl_3_ at 400 MHz, Figure S8: HRESIMS spectrum of **2**, Figure S9: ^1^H NMR spectrum of **2** in CDCl_3_ at 500 MHz, Figure S10: ^13^C NMR spectrum of **2** in CDCl_3_ at 125 MHz, Figure S11: HSQC NMR spectrum of **2** in CDCl_3_ at 500 MHz, Figure S12: COSY NMR spectrum of **2** in CDCl_3_ at 500 MHz, Figure S13: HMBC NMR spectrum of **2** in CDCl_3_ at 500 MHz, Figure S14: NOESY NMR spectrum of **2** in CDCl_3_ at 500 MHz, Figure S15: HRESIMS spectrum of **3**, Figure S16: ^1^H NMR spectrum of **3** in CDCl_3_ at 400 MHz, Figure S17: ^13^C NMR spectrum of **3** in CDCl_3_ at 100 MHz, Figure S18: HSQC NMR spectrum of **3** in CDCl_3_ at 400 MHz, Figure S19: COSY NMR spectrum of **3** in CDCl_3_ at 400 MHz, Figure S20: HMBC NMR spectrum of **3** in CDCl_3_ at 400 MHz, Figure S21: NOESY NMR spectrum of **3** in CDCl_3_ at 400 MHz, Figure S22: ^1^H NMR spectrum of **3** in CD_3_OD at 400 MHz, Figure S23: ^13^C NMR spectrum of **3** in CD_3_OD at 100 MHz, Figure S24: HSQC NMR spectrum of **3** in CD_3_OD at 400 MHz, Figure S25: COSY NMR spectrum of **3** in CD_3_OD at 400 MHz, Figure S26: HMBC NMR spectrum of **3** in CD_3_OD at 400 MHz.
