# Supplementary material for: Anti-Inflammatory Polyoxygenated Steroids from the Soft Coral Lobophytum michaelae
Source: Mar Drugs. 2018 Mar 13;16(3):93. doi: 10.3390/md16030093 (PMC5867637; doi:10.3390/md16030093)
Supplement: Supplementary file 1 [file marinedrugs-16-00093-s001.pdf]

Supplementary Materials

Anti-inflammatory Polyoxygenated Steroids from the Soft Coral *Lobophytum michaelae*

Chiung-Yao Huang [1,†], Wan-Ru Tseng [1,†], Atallah F. Ahmed [2], Pei-Lun Chiang [3], Chi-Jen Tai [4], Tsong-Long Hwang [5,6,7], Chang-Feng Dai [8] and Jyh-Horng Sheu [1,9,10,11,*]

[1] Department of Marine Biotechnology and Resources, National Sun Yat-sen University, Kaohsiung 804, Taiwan
[2] Department of Pharmacognosy, College of Pharmacy, King Saud University, Riyadh 11451, Saudi Arabia
[3] Department of Biochemistry, University of Toronto, Toronto M5G2R3, Canada
[4] Doctoral Degree Program in Marine Biotechnology, National Sun Yat-sen University and Academia Sinica, Kaohsiung 804, Taiwan
[5] Graduate Institute of Natural Products, College of Medicine, Chang Gung University, Taoyuan 333, Taiwan
[6] Research Center for Chinese Herbal Medicine, Research Center for Food and Cosmetic Safety, and Graduate Institute of Health Industry Technology, College of Human Ecology, Chang Gung University of Science and Technology, Taoyuan 333, Taiwan
[7] Department of Anesthesiology, Chang Gung Memorial Hospital, Taoyuan 333, Taiwan
[8] Institute of Oceanography, National Taiwan University, Taipei 112, Taiwan
[9] Graduate Institute of Natural Products, Kaohsiung Medical University, Kaohsiung 807, Taiwan
[10] Department of Medical Research, China Medical University Hospital, China Medical University, Taichung 404, Taiwan
[11] Frontier Center for Ocean Science and Technology, National Sun Yat-sen University, Kaohsiung 804, Taiwan

[†]These authors contributed equally to this work.

Corresponding author: sheu@mail.nsysu.edu.tw

List of Supplementary Materials:

No	Content	page	No	Content	page
**Figure S1**	HRESIMS spectrum of **1**.	3	**Figure S14**	NOESY NMR spectrum of **2** in $CDCl_3$ at 500 MHz	16
**Figure S2**	^{1}H NMR spectrum of **1** in $CDCl_3$ at 400 MHz.	4	**Figure S15**	HRESIMS spectrum of **3**	17
**Figure S3**	^{13}C NMR spectrum of **1** in $CDCl_3$ at 100 MHz.	5	**Figure S16**	^{1}H NMR spectrum of **3** in $CDCl_3$ at 400 MHz	18
**Figure S4**	HSQC NMR spectrum of **1** in $CDCl_3$ at 400 MHz	6	**Figure S17**	^{13}C NMR spectrum of **3** in $CDCl_3$ at 100 MHz	19
**Figure S5**	COSY NMR spectrum of **1** in $CDCl_3$ at 400 MHz	7	**Figure S18**	HSQC NMR spectrum of **3** in $CDCl_3$ at 400 MHz	20
**Figure S6**	HMBC NMR spectrum of **1** in $CDCl_3$ at 400 MHz	8	**Figure S19**	COSY NMR spectrum of **3** in $CDCl_3$ at 400 MHz	21
**Figure S7**	NOESY NMR spectrum of **1** in $CDCl_3$ at 400 MHz	9	**Figure S20**	HMBC NMR spectrum of **3** in $CDCl_3$ at 400 MHz	22
**Figure S8**	HRESIMS spectrum of **2**	10	**Figure S21**	NOESY NMR spectrum of **3** in $CDCl_3$ at 400 MHz	23
**Figure S9**	^{1}H NMR spectrum of **2** in $CDCl_3$ at 500 MHz	11	**Figure S22**	^{1}H NMR spectrum of **3** in CD_3OD at 400 MHz	24
**Figure S10**	^{13}C NMR spectrum of **2** in $CDCl_3$ at 125 MHz	12	**Figure S23**	^{13}C NMR spectrum of **3** in CD_3OD at 100 MHz	25
**Figure S11**	HSQC NMR spectrum of **2** in $CDCl_3$ at 500 MHz	13	**Figure S24**	HSQC NMR spectrum of **3** in CD_3OD at 400 MHz	26
**Figure S12**	COSY NMR spectrum of **2** in $CDCl_3$ at 500 MHz	14	**Figure S25**	COSY NMR spectrum of **3** in CD_3OD at 400 MHz	27
**Figure S13**	HMBC NMR spectrum of **2** in $CDCl_3$ at 500 MHz	15	**Figure S26**	HMBC NMR spectrum of **3** in CD_3OD at 400 MHz	28

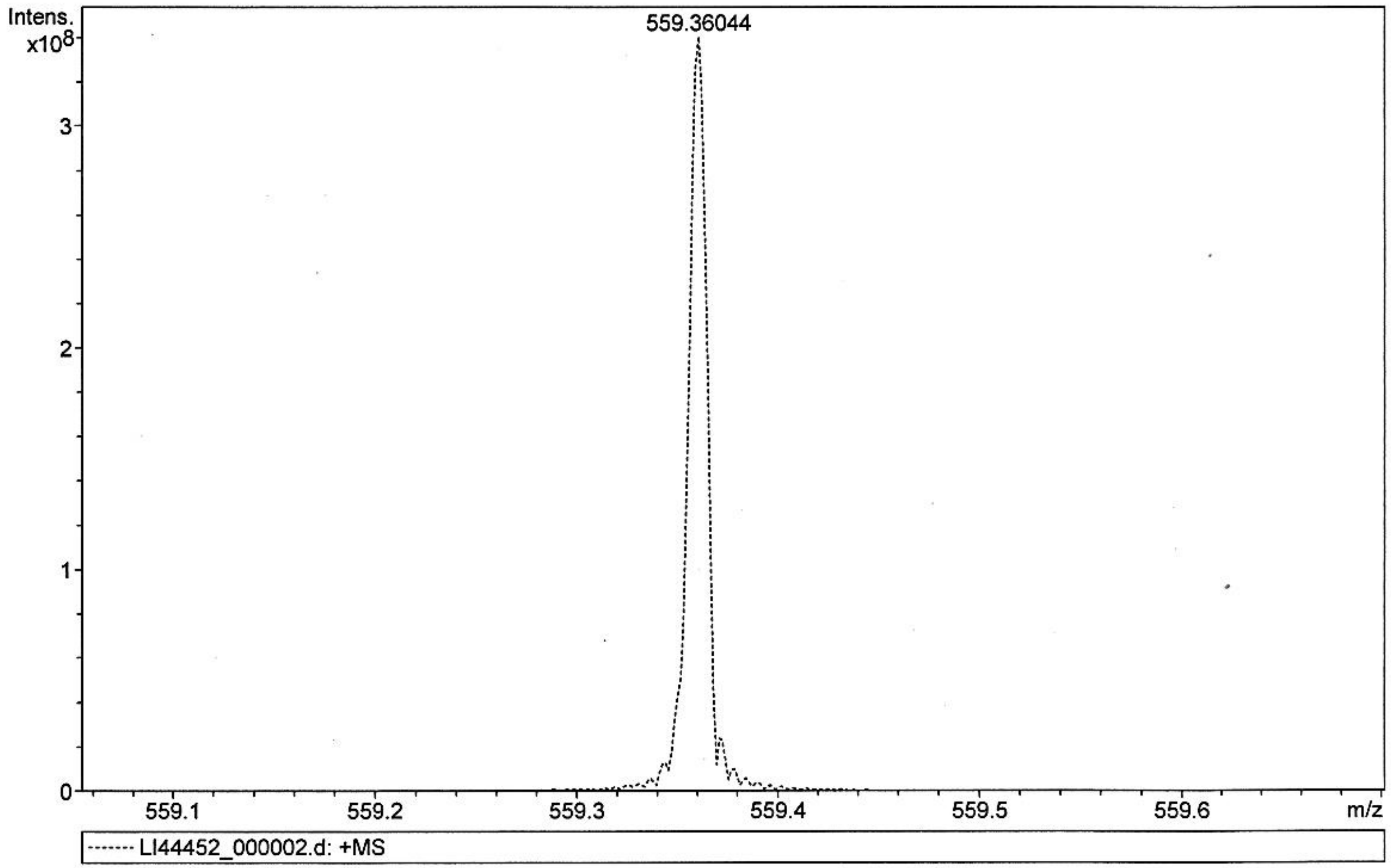

Meas. m/z	#	Formula	Score	m/z	err [mDa]	err [ppm]	mSigma	rdb	e⁻ Conf	N-Rule
559.36044	1	C 31 H 52 Na O 7	100.00	559.36053	0.08	0.15	15.6	5.5	even	ok

Figure S1. HRESIMS spectrum of **1**.

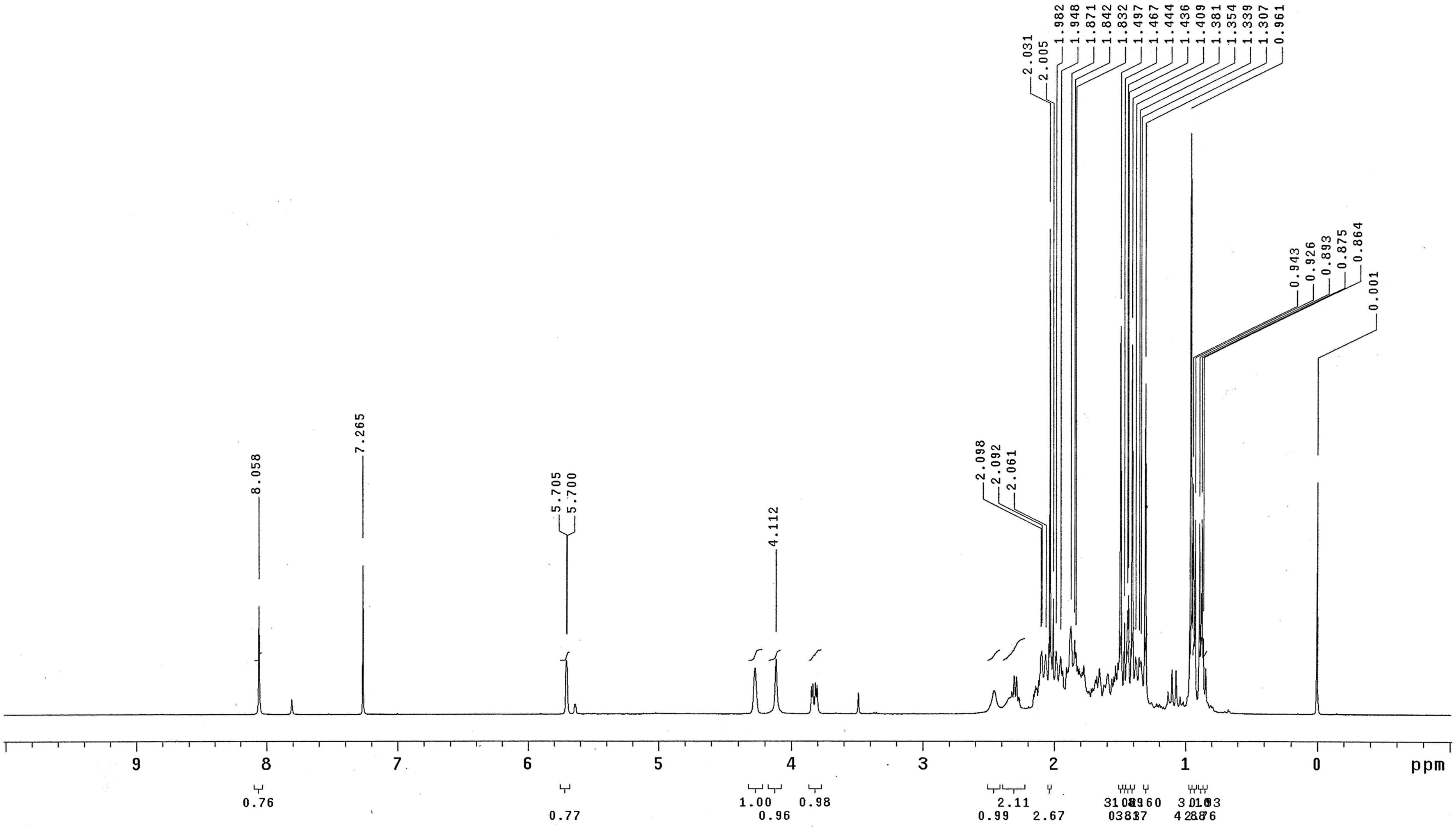

Figure S2. ^{1}H NMR spectrum of **1** in $CDCl_3$ at 400 MHz.

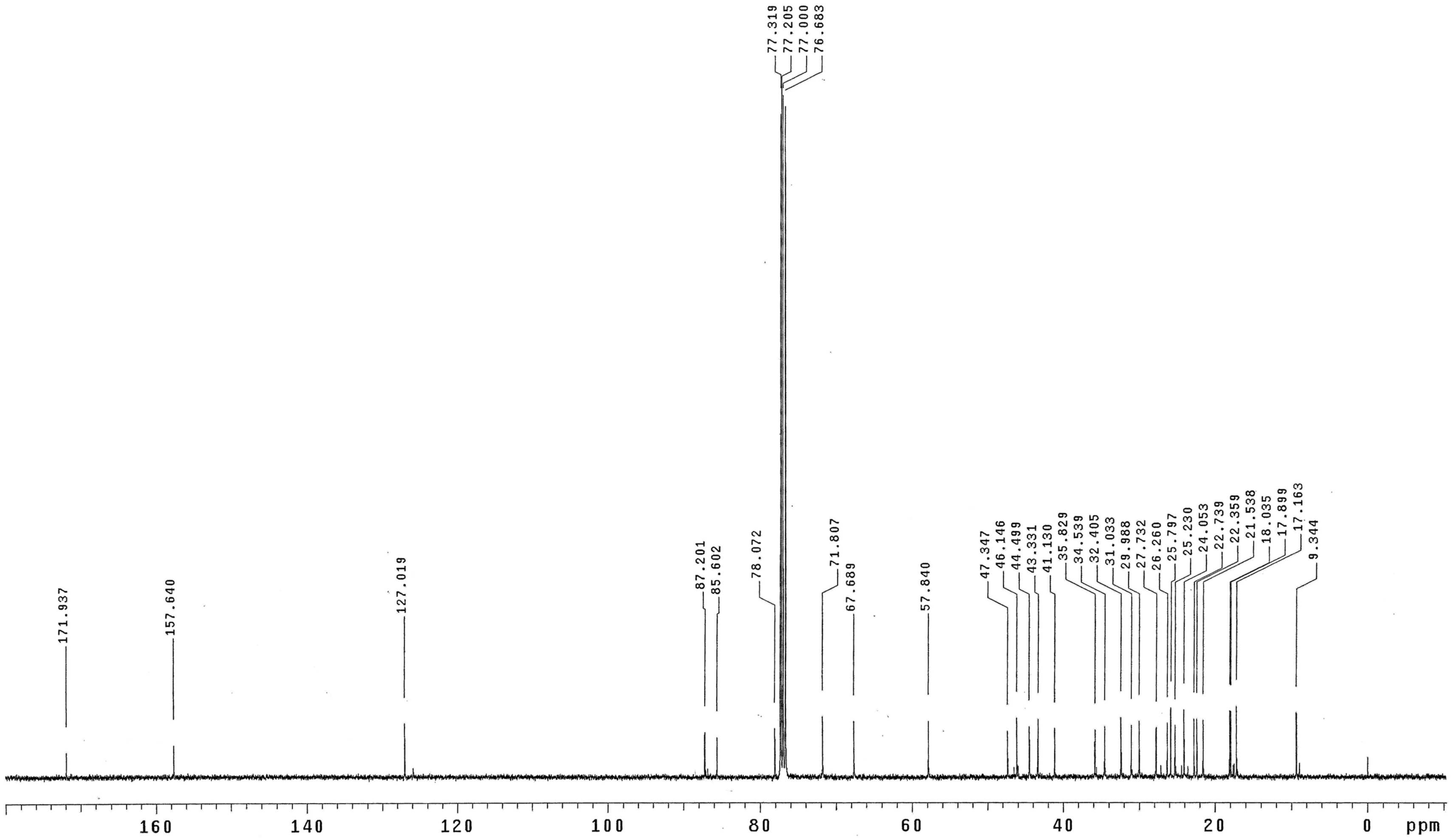

Figure S3. ^{13}C NMR spectrum of **1** in $CDCl_3$ at 100 MHz.

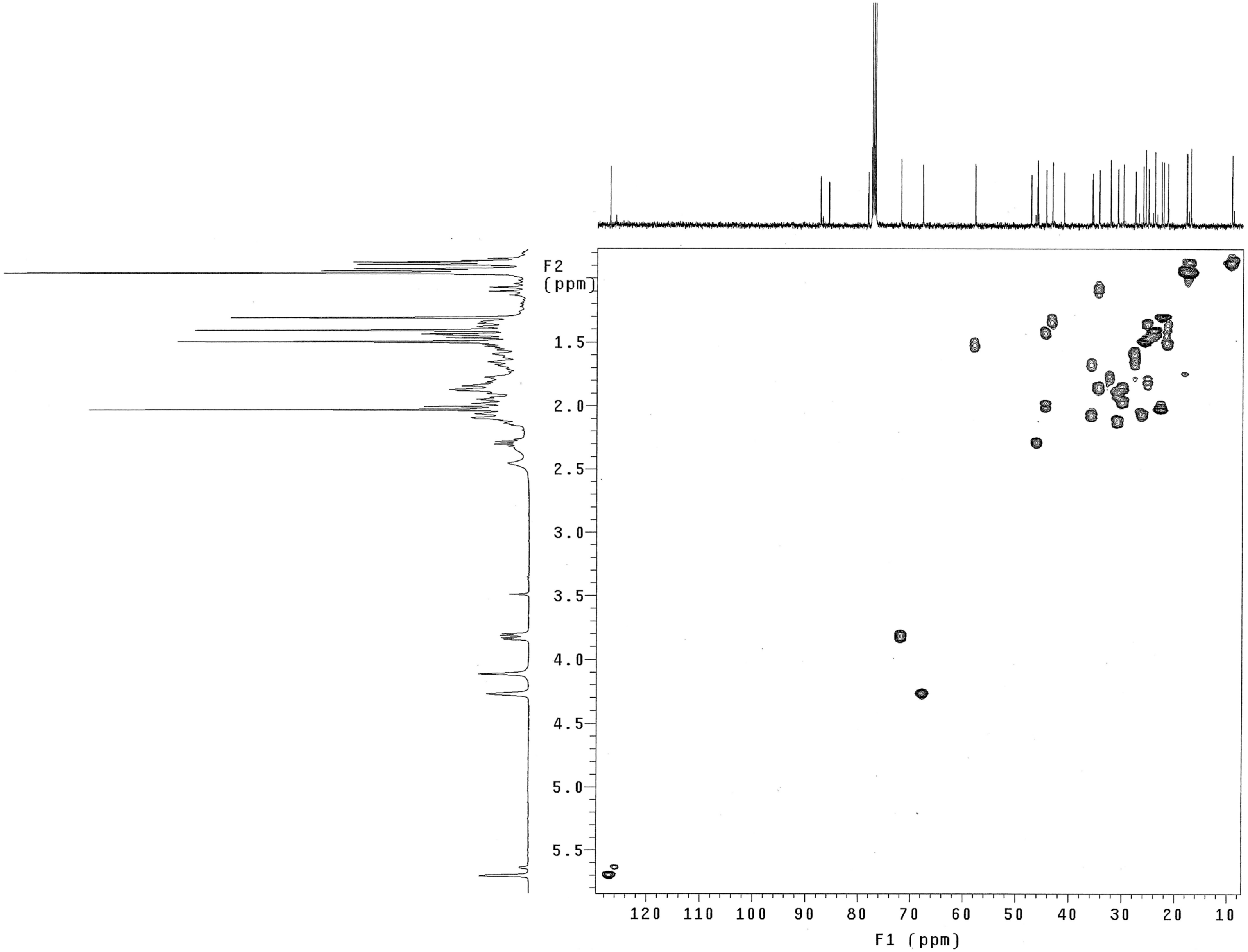

Figure S4. HSQC NMR spectrum of **1** in $CDCl_3$ at 400 MHz.

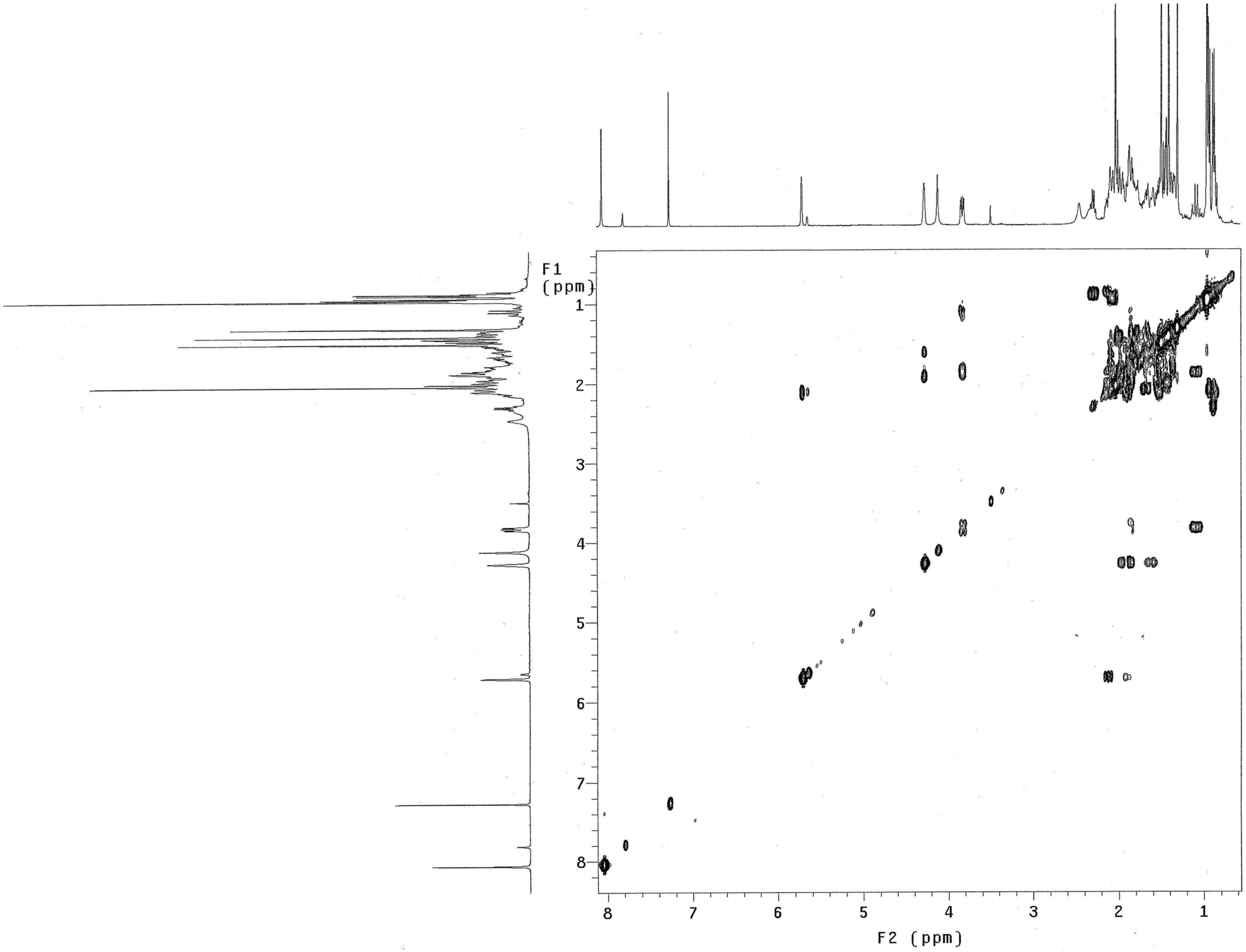

Figure S5. COSY NMR spectrum of **1** in $CDCl_3$ at 400 MHz.

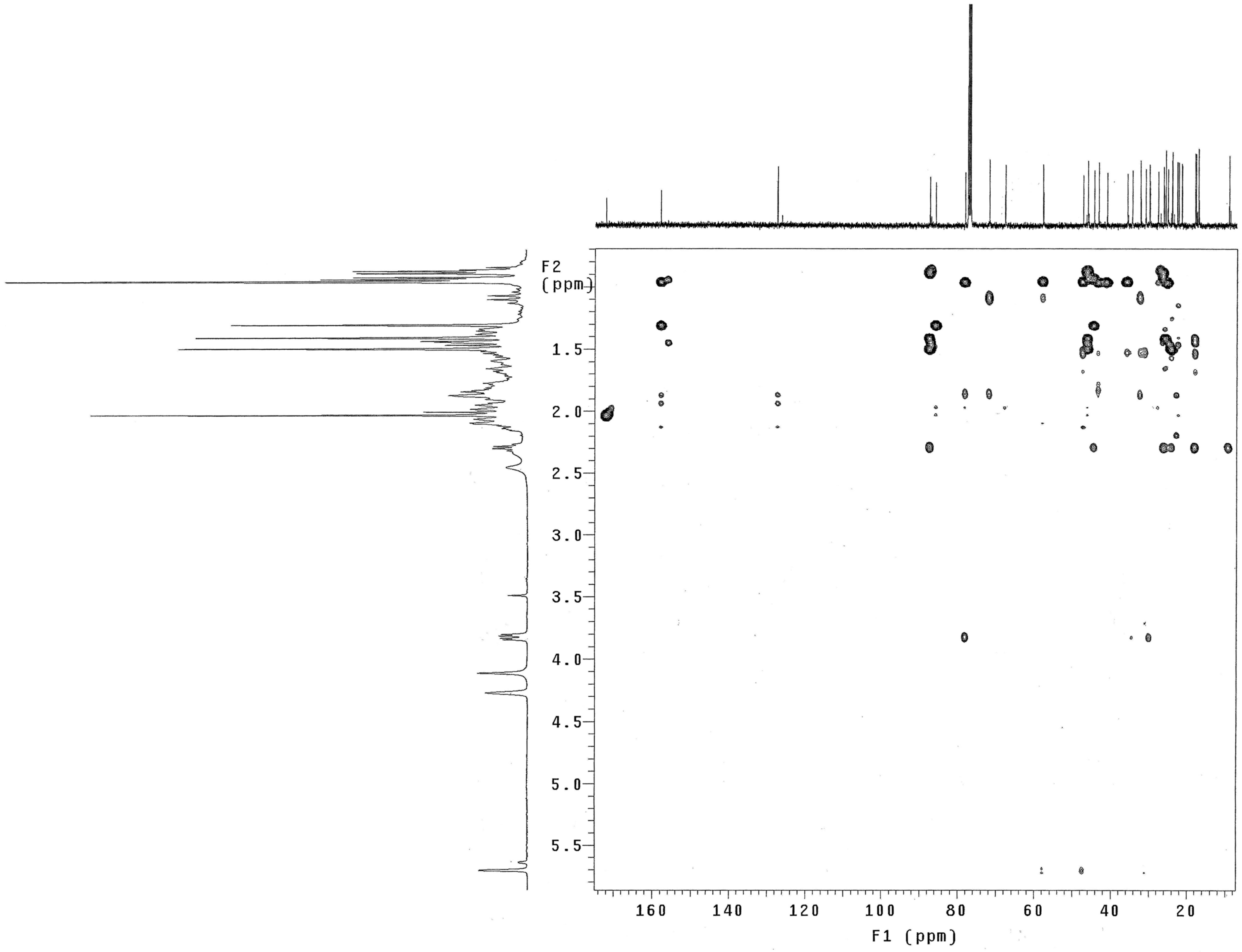

Figure S6. HMBC NMR spectrum of **1** in $CDCl_3$ at 400 MHz.

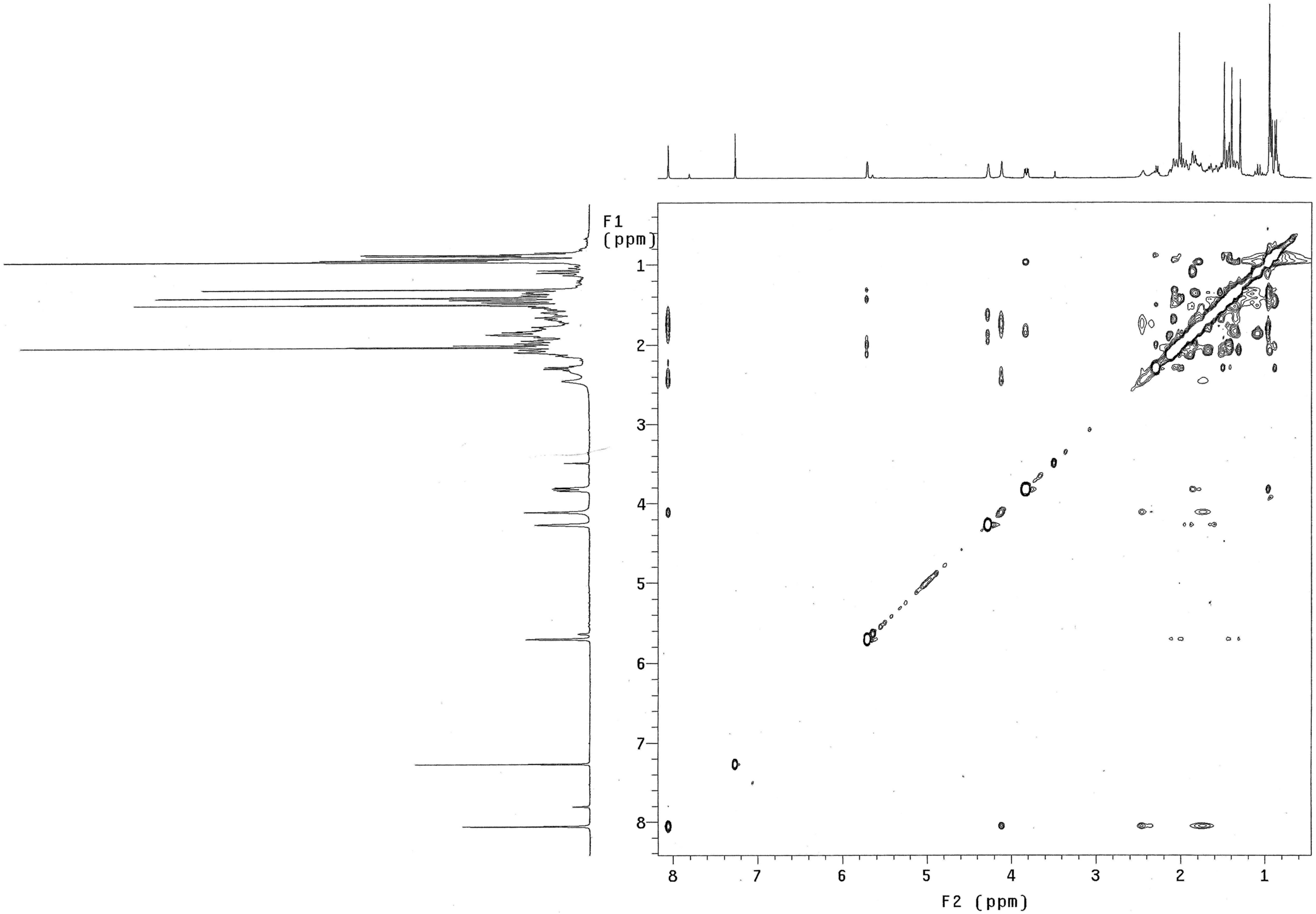

Figure S7. NOESY NMR spectrum of **1** in $CDCl_3$ at 400 MHz.

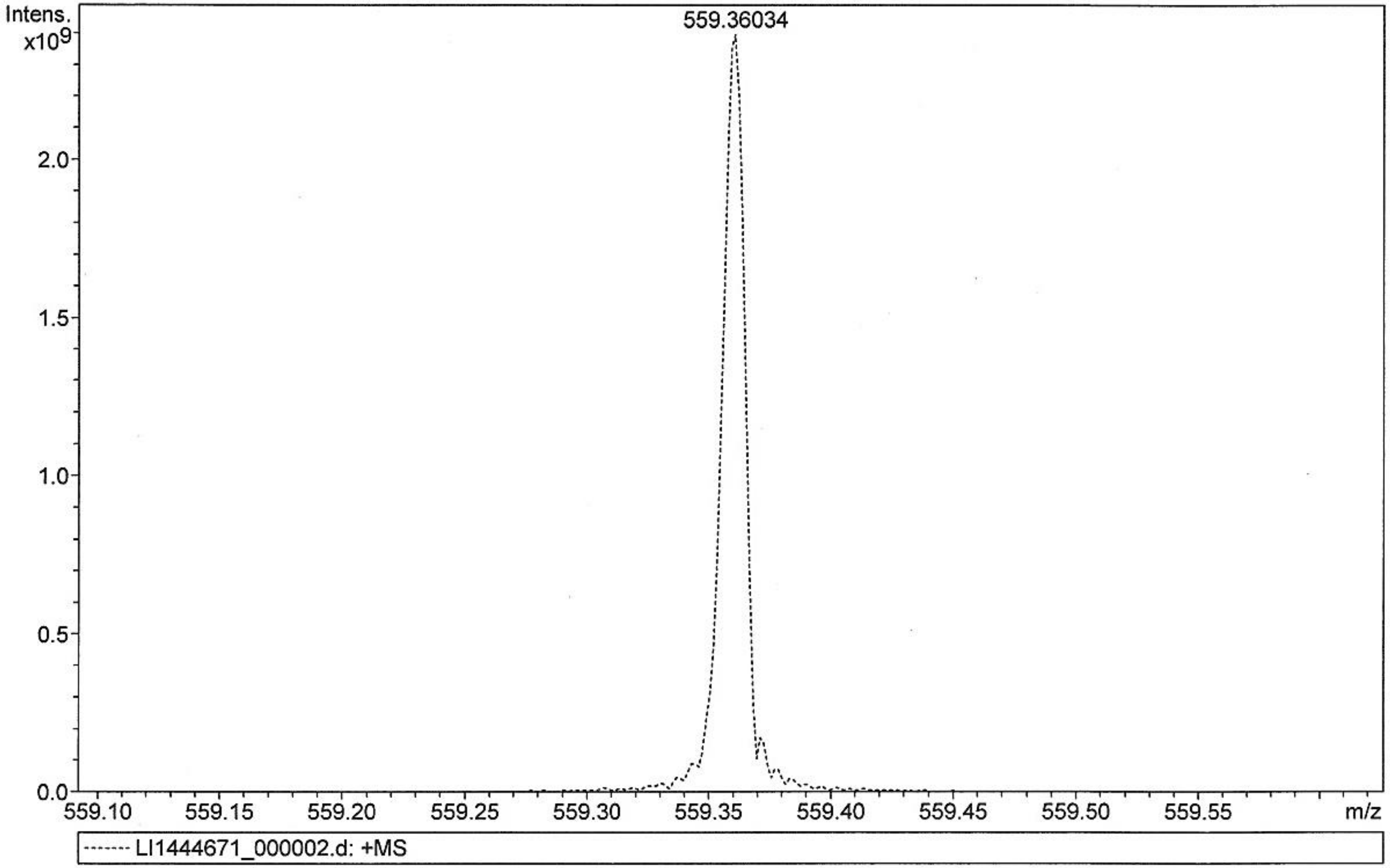

Meas. m/z	#	Formula	Score	m/z	err [mDa]	err [ppm]	mSigma	rdb	e⁻ Conf	N-Rule
559.36034	1	C 31 H 52 Na O 7	100.00	559.36053	0.19	0.33	14.6	5.5	even	ok

Figure S8. HRESIMS spectrum of **2**.

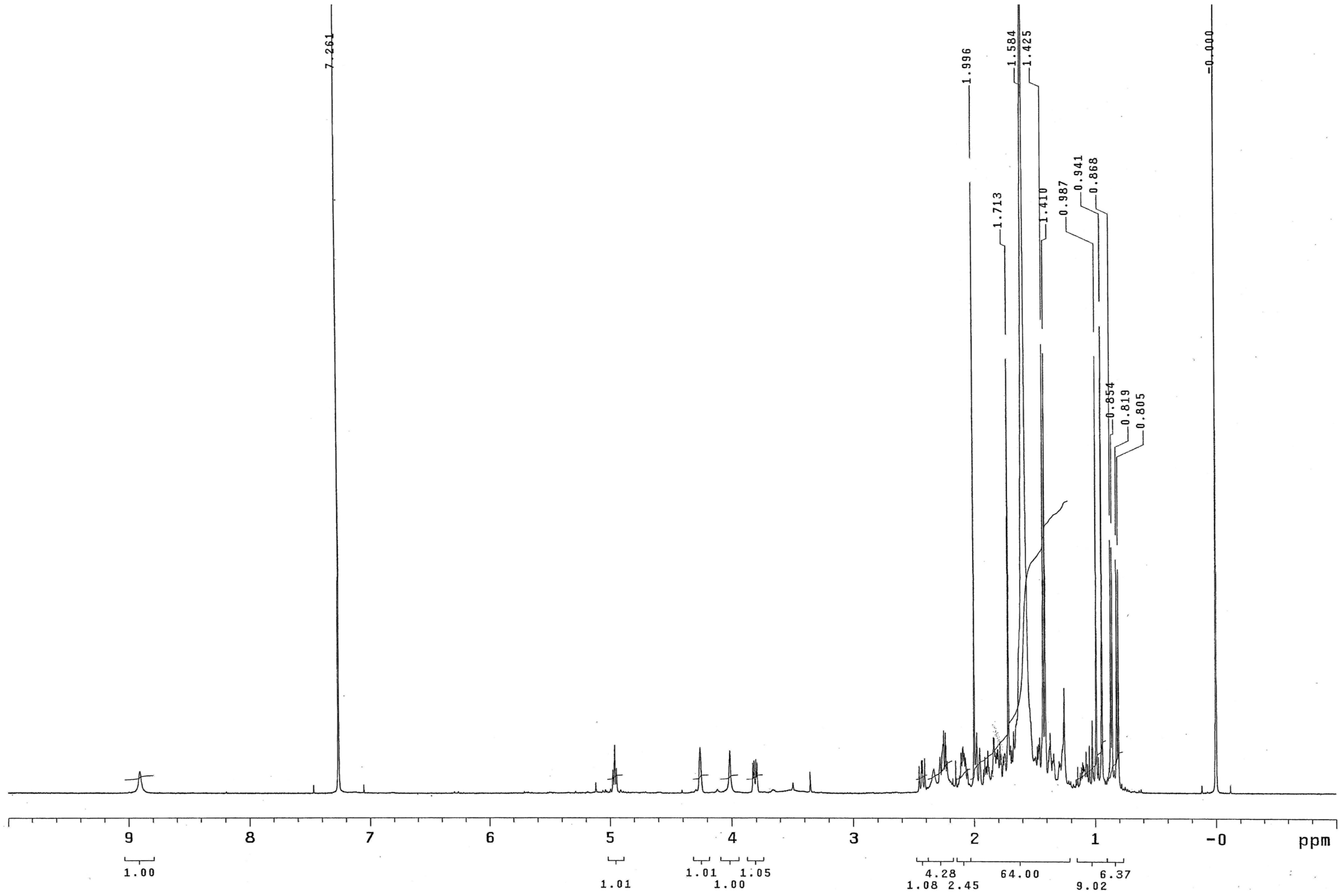

Figure S9. ^{1}H NMR spectrum of **2** in $CDCl_3$ at 500 MHz.

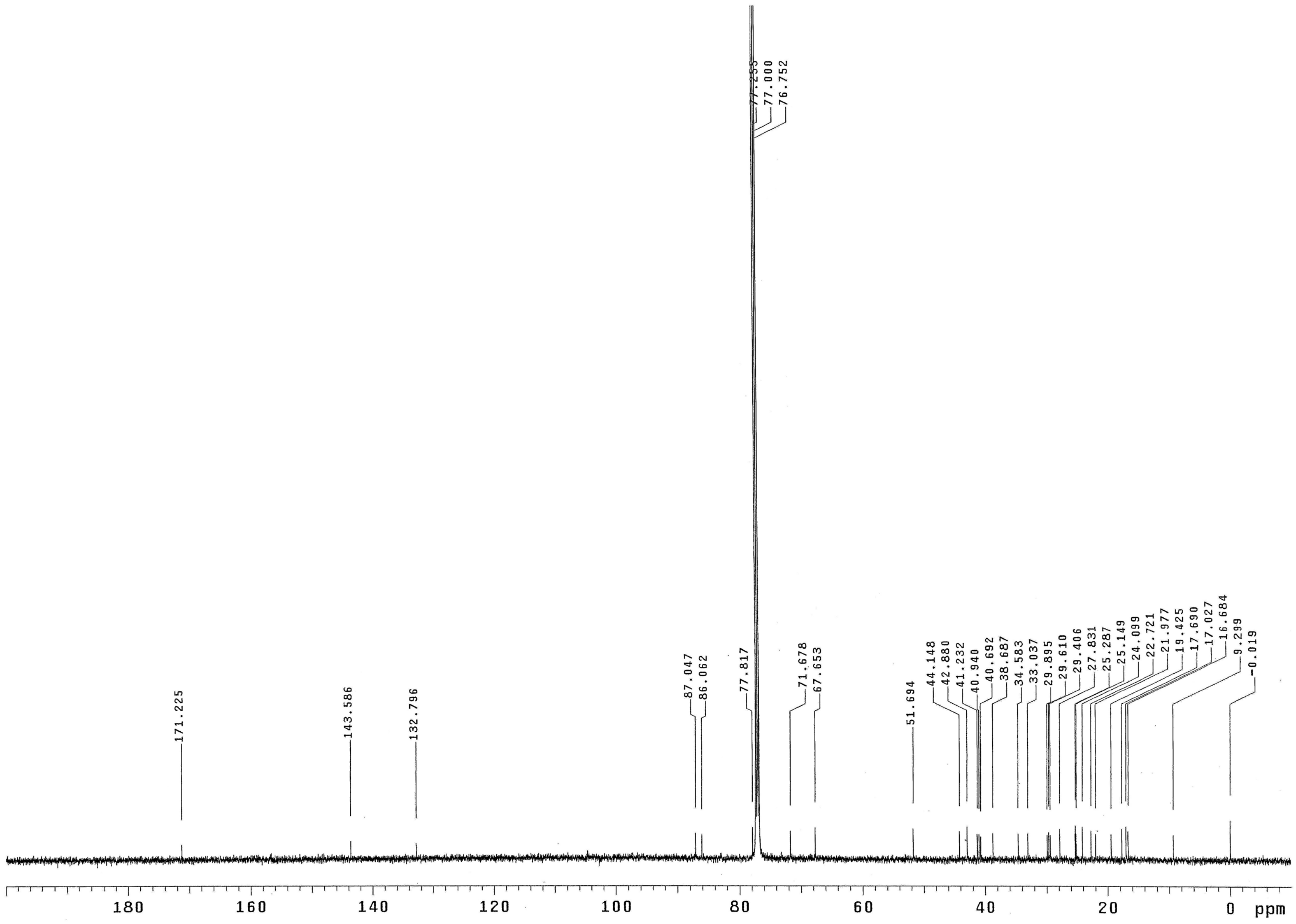

Figure S10. ^{13}C NMR spectrum of **2** in $CDCl_3$ at 125 MHz.

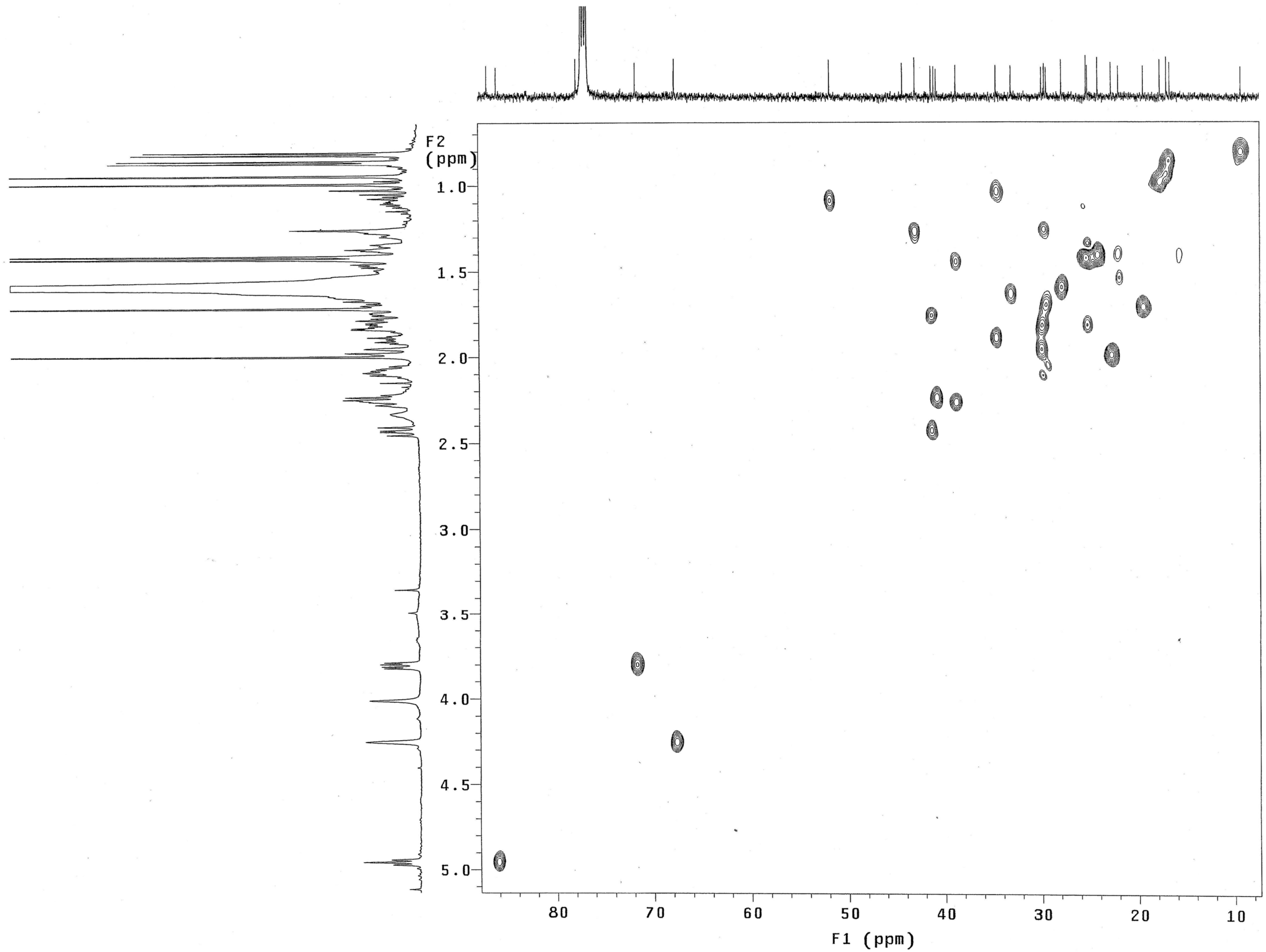

Figure S11. HSQC NMR spectrum of **2** in $CDCl_3$ at 500 MHz.

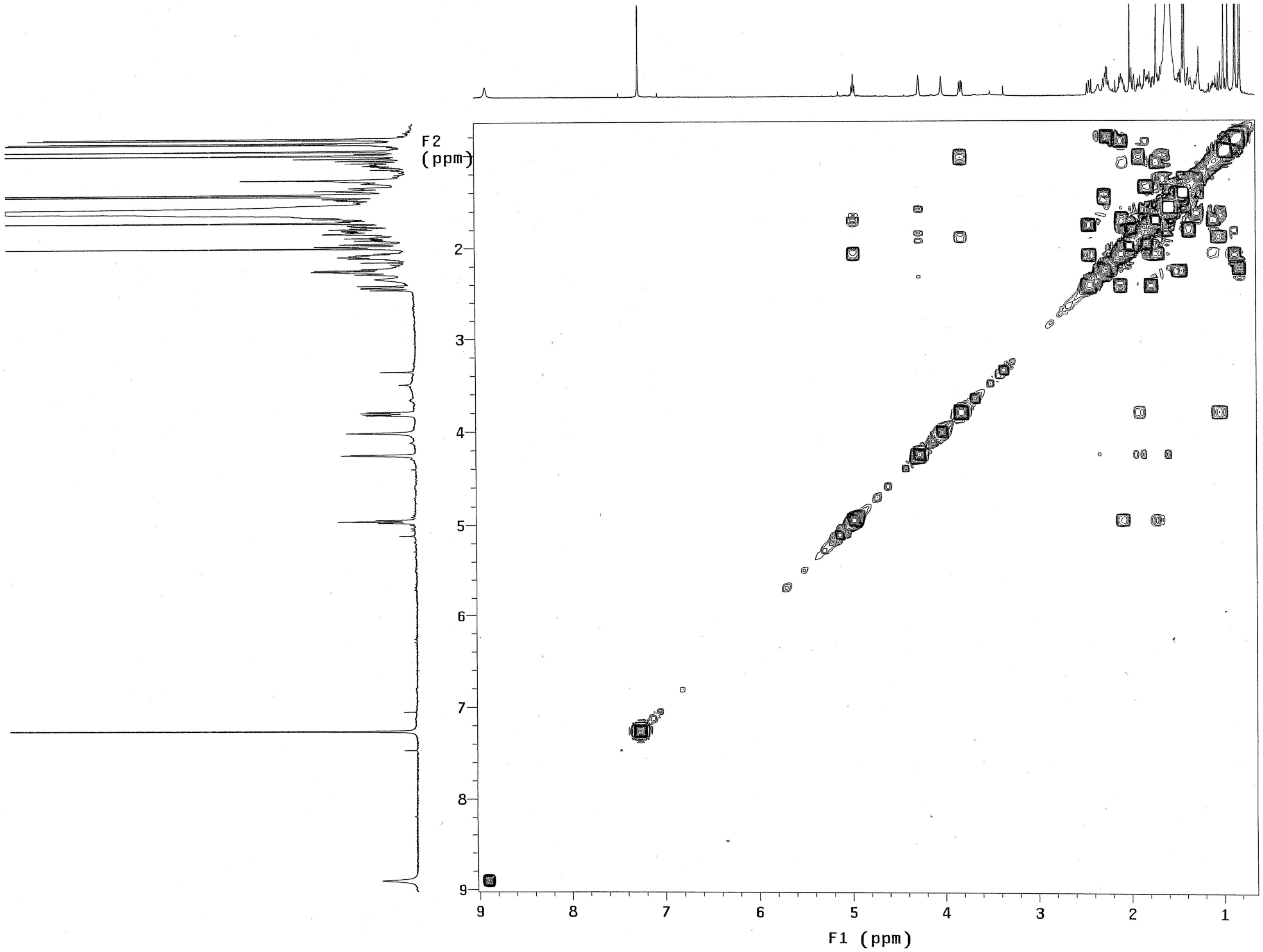

Figure S12. COSY NMR spectrum of **2** in $CDCl_3$ at 500 MHz.

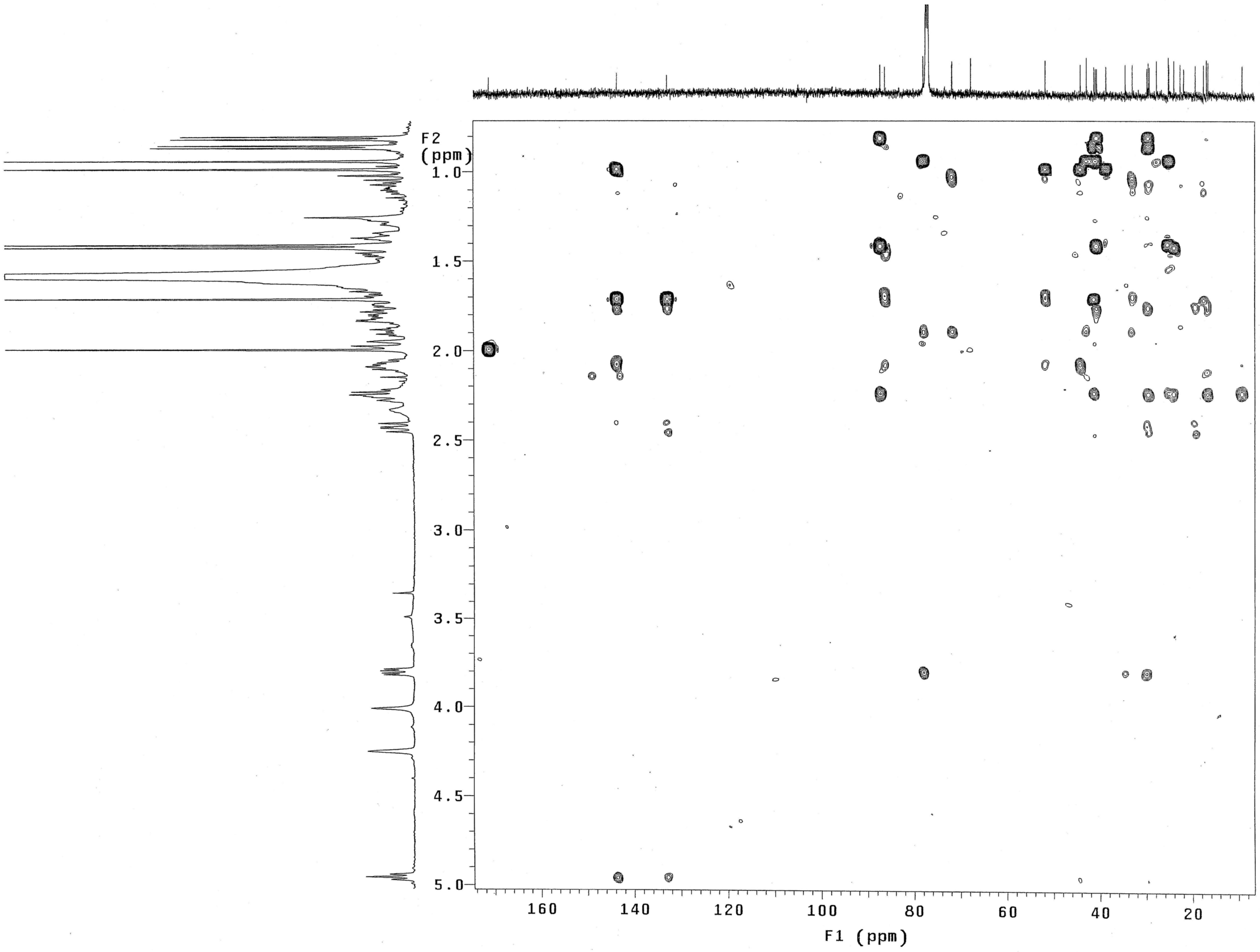

Figure S13. HMBC NMR spectrum of **2** in $CDCl_3$ at 500 MHz.

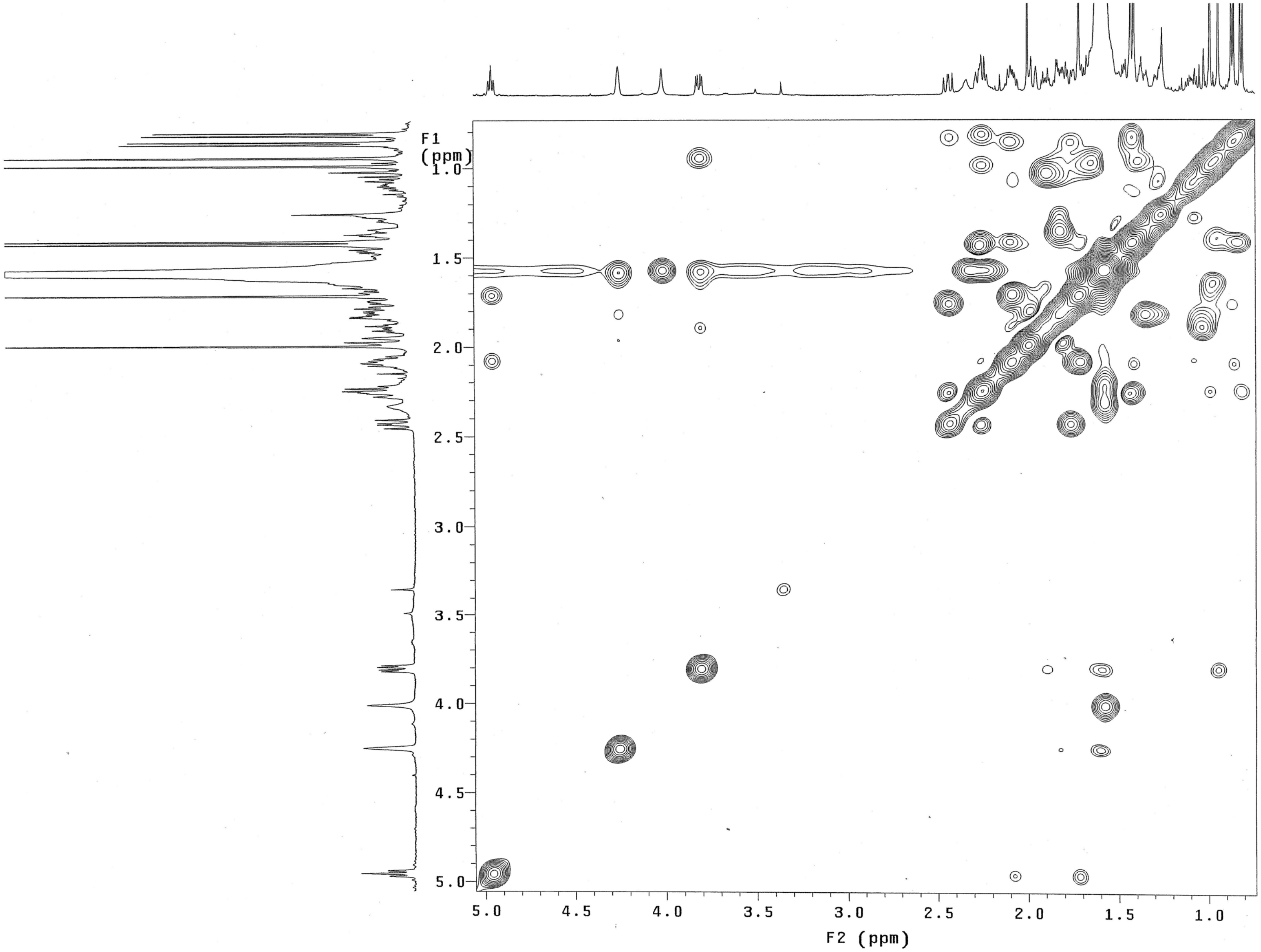

Figure S14. NOESY NMR spectrum of **2** in $CDCl_3$ at 500 MHz.

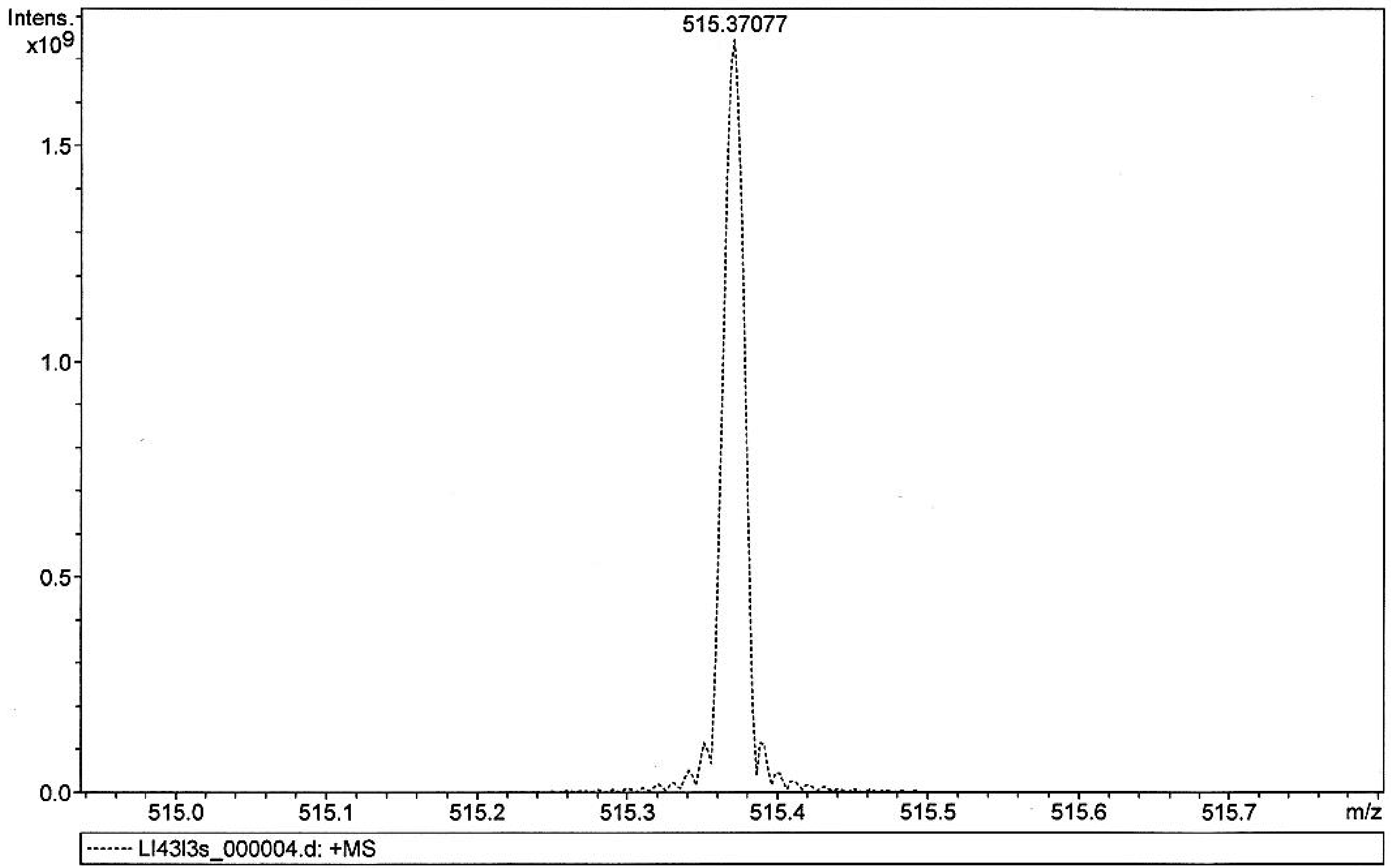

Meas. m/z	#	Formula	Score	m/z	err [mDa]	err [ppm]	mSigma	rdb	e^- Conf	N-Rule
515.37077	1	C 30 H 52 Na O 5	100.00	515.37070	-0.08	-0.15	3.7	4.5	even	ok

Figure S15. HRESIMS spectrum of **3**.

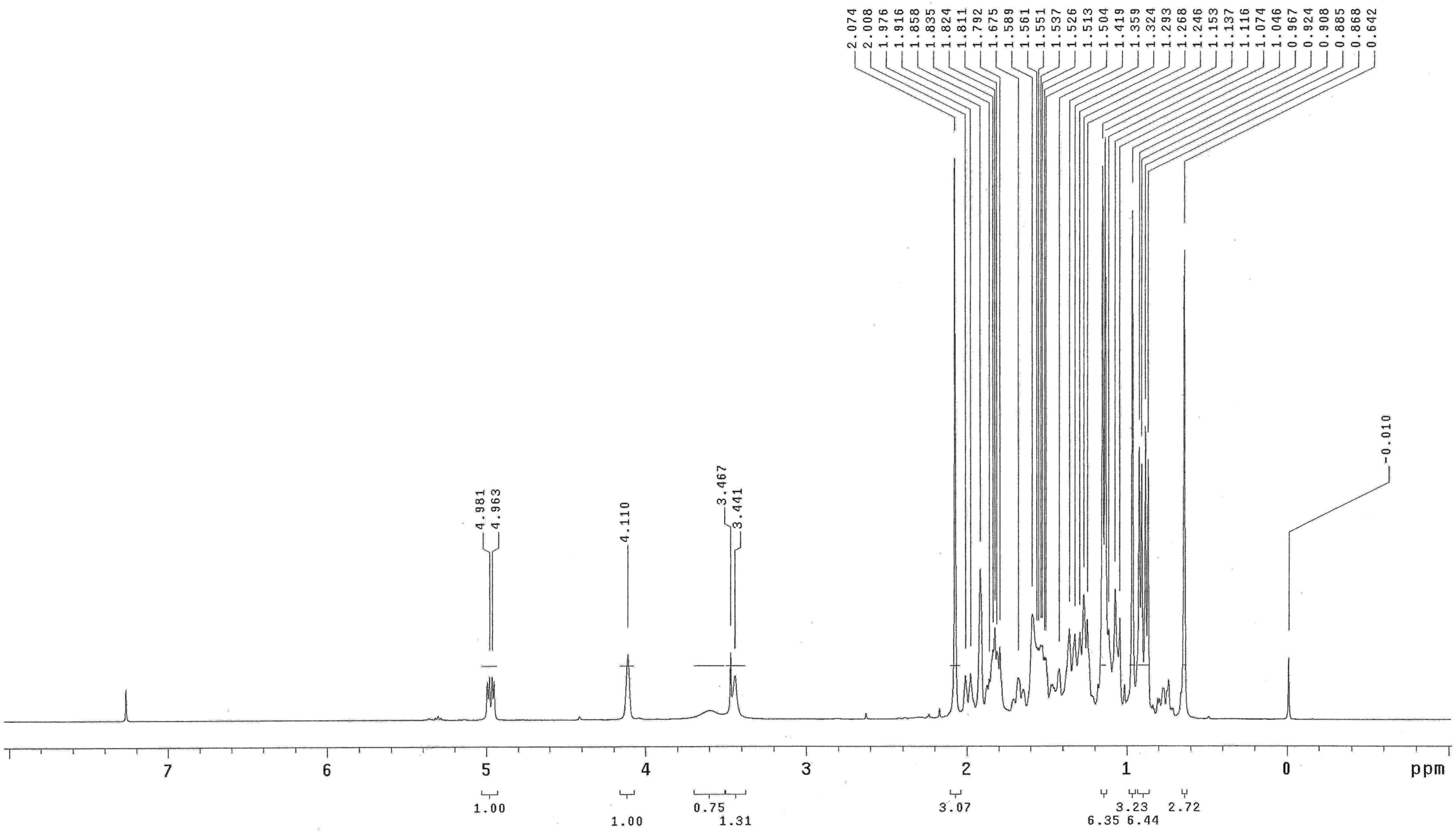

Figure S16. ^{1}H NMR spectrum of **3** in $CDCl_3$ at 400 MHz.

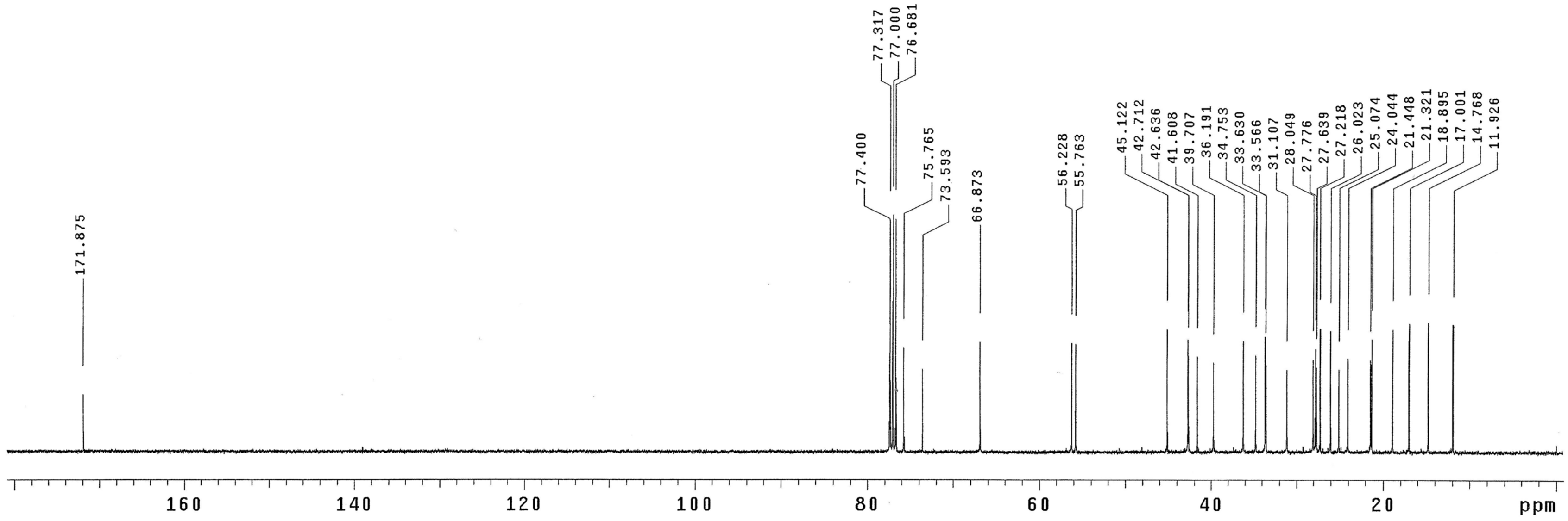

Figure S17. ^{13}C NMR spectrum of **3** in $CDCl_3$ at 100 MHz.

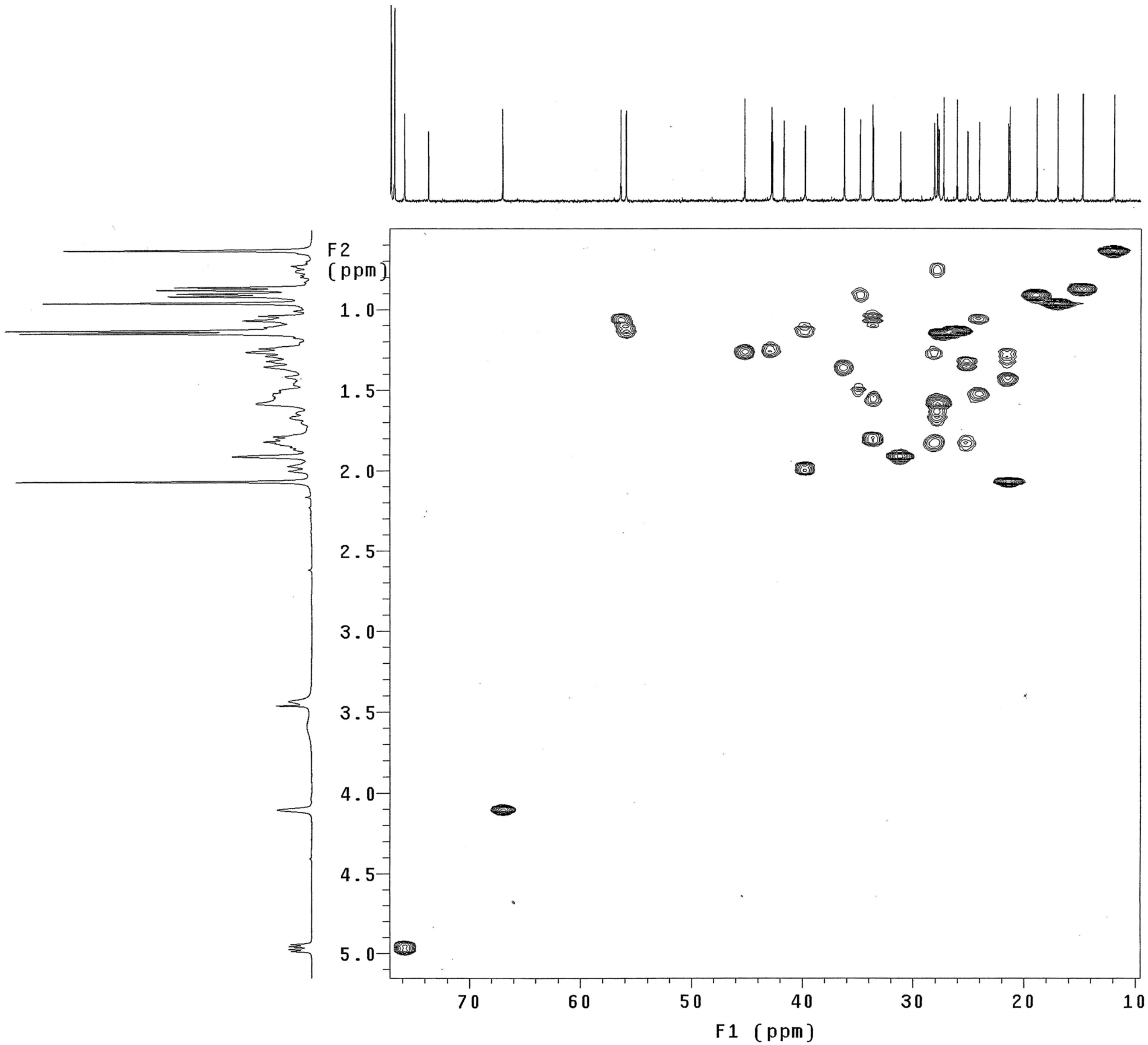

Figure S18. HSQC NMR spectrum of **3** in $CDCl_3$ at 400 MHz.

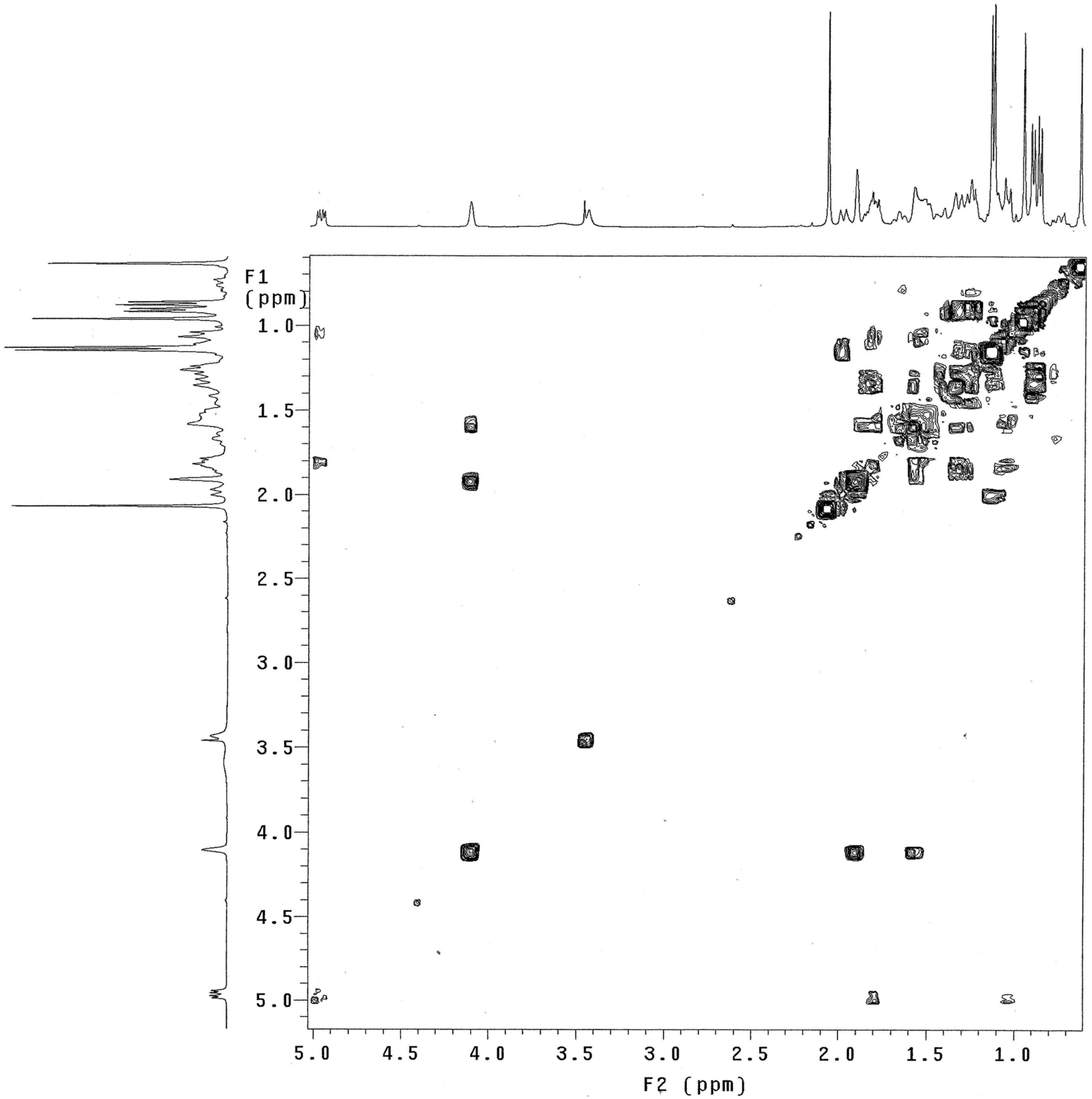

Figure S19. COSY NMR spectrum of **3** in $CDCl_3$ at 400 MHz.

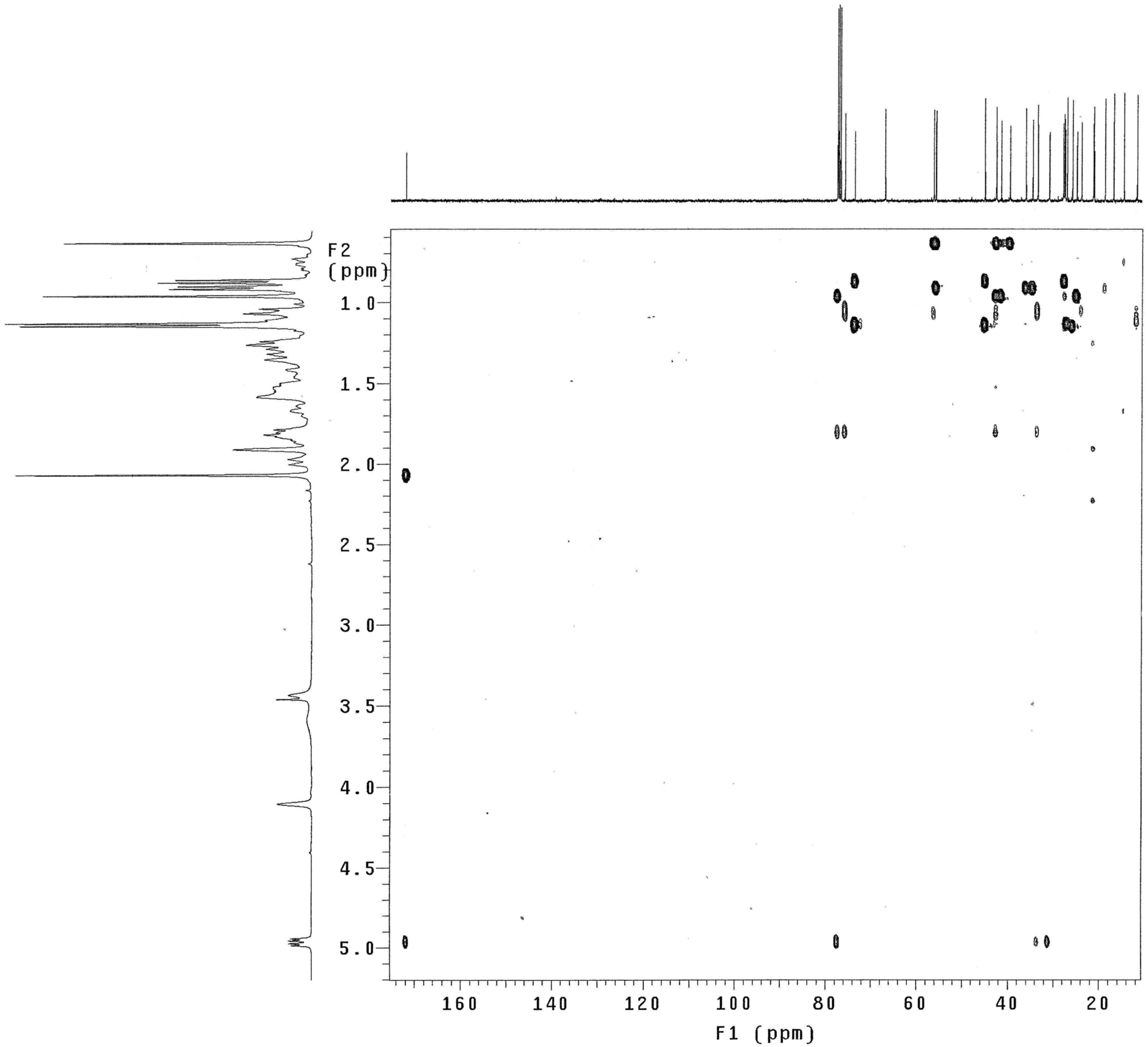

Figure S20. HMBC NMR spectrum of **3** in $CDCl_3$ at 400 MHz.

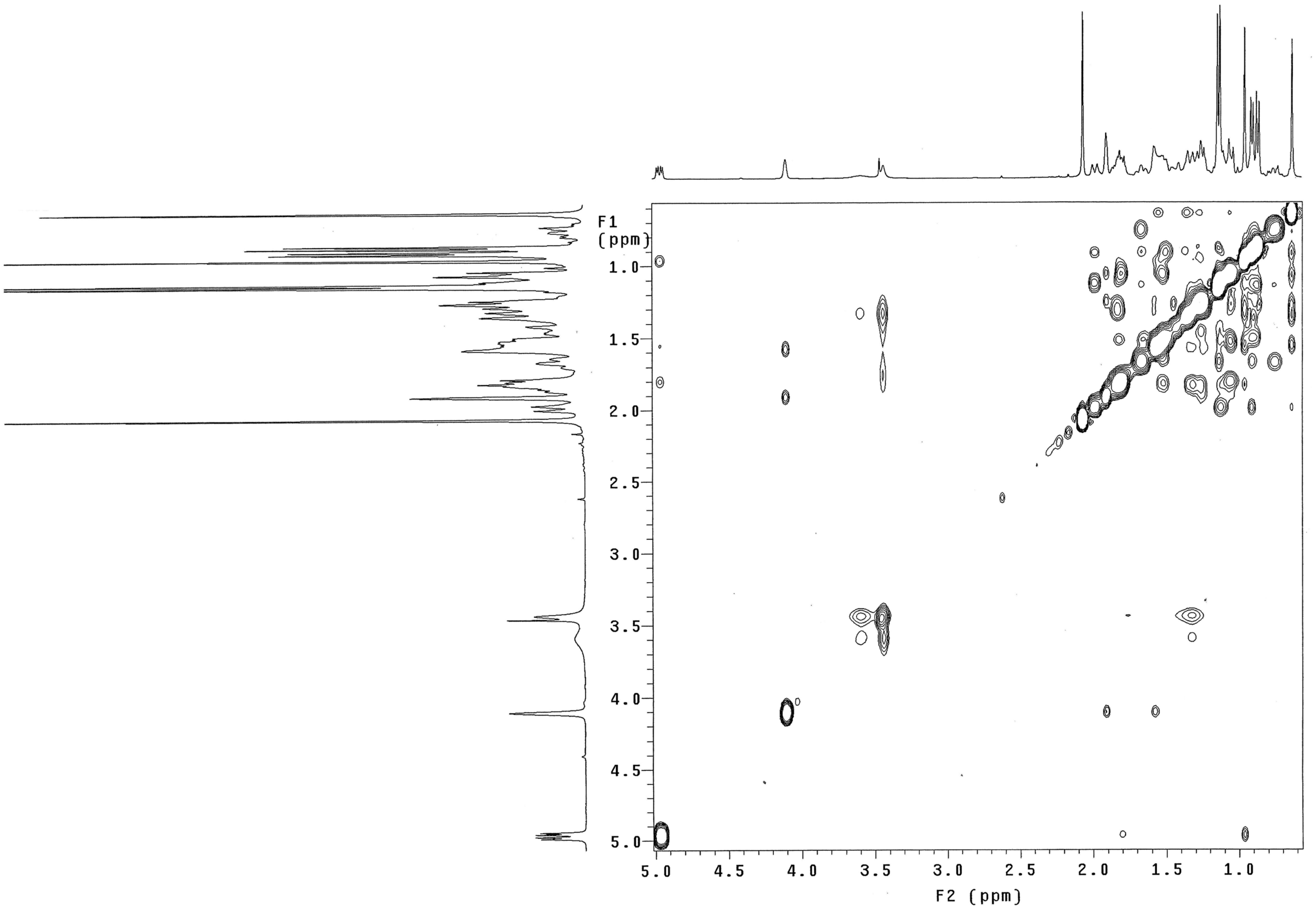

Figure S21. NOESY NMR spectrum of **3** in $CDCl_3$ at 400 MHz.

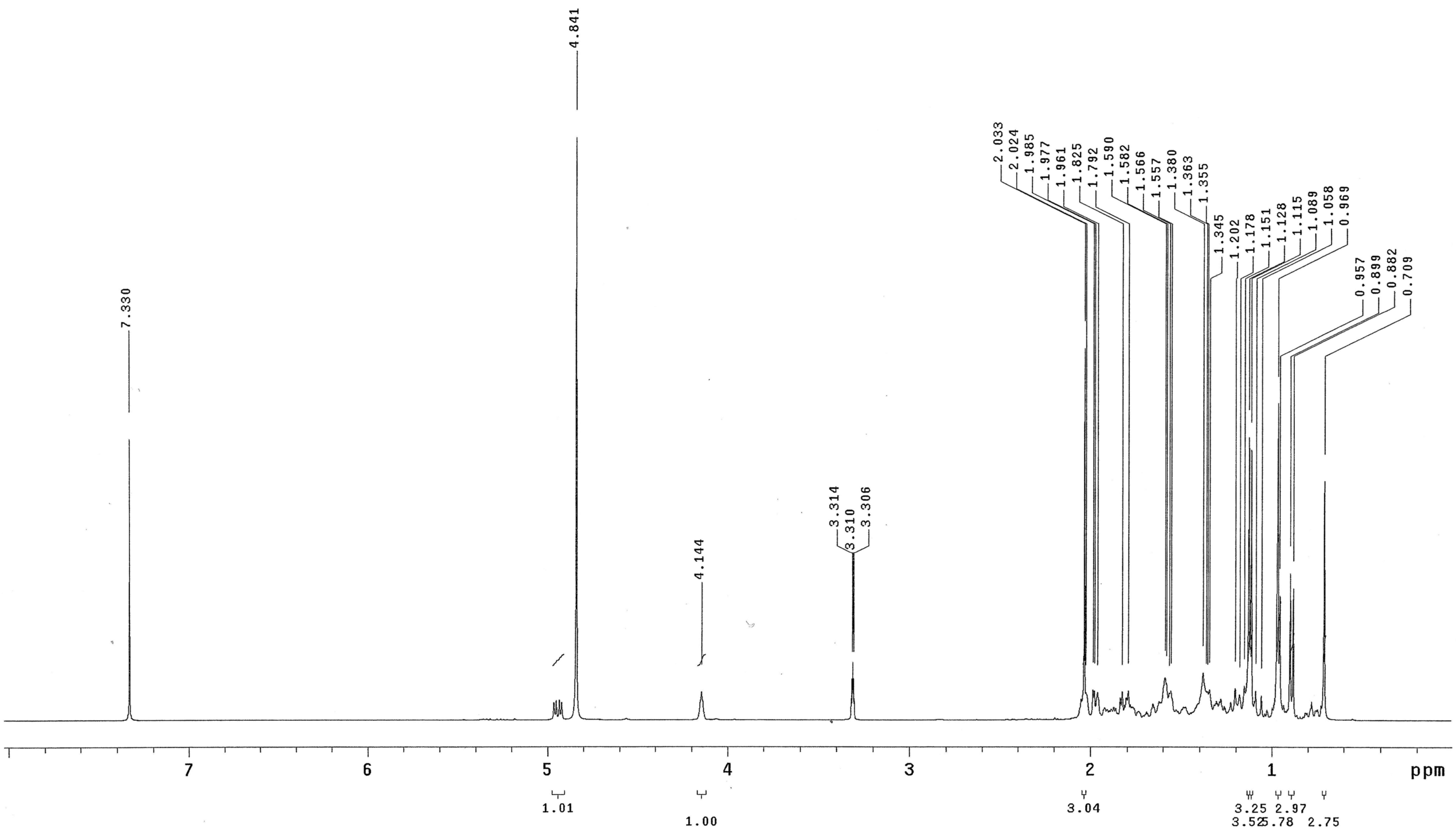

Figure S22. ^{1}H NMR spectrum of **3** in CD_3OD at 400 MHz.

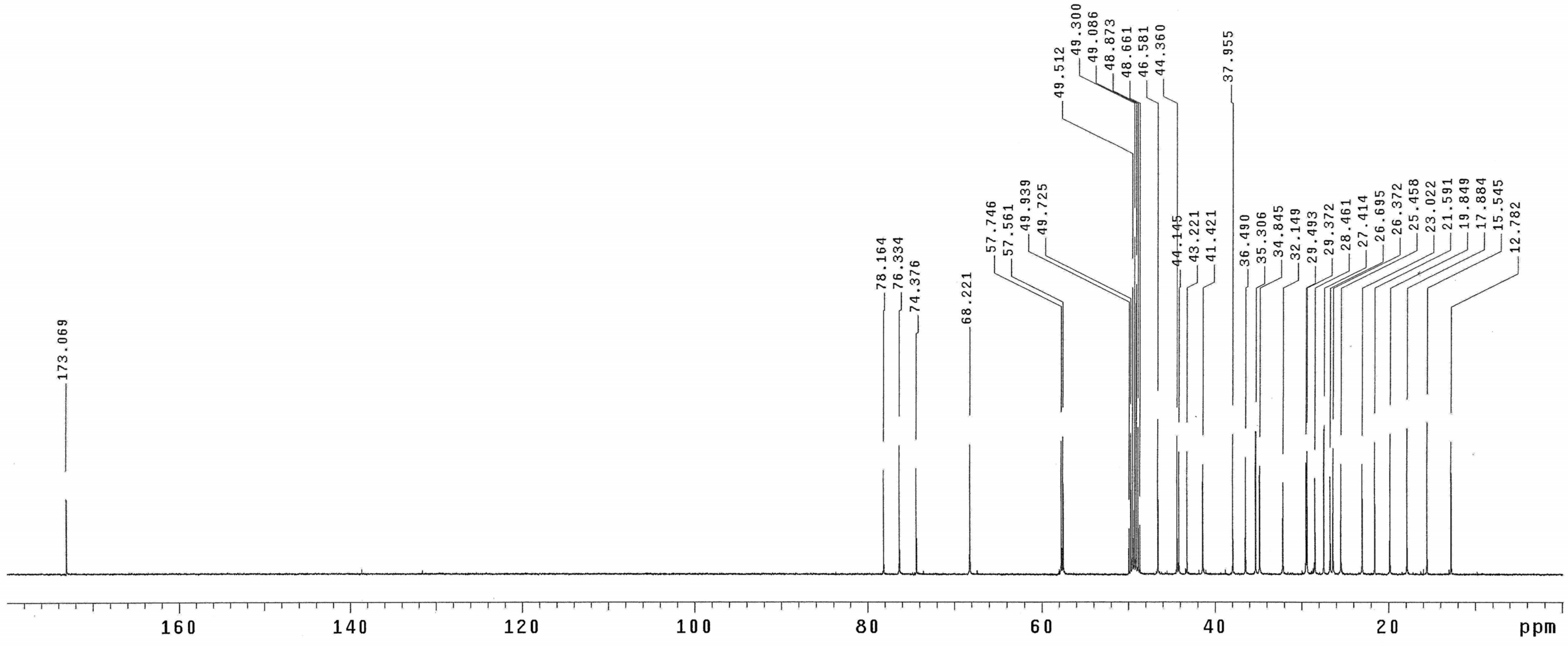

Figure S23. ^{13}C NMR spectrum of **3** in CD_3OD at 100 MHz.

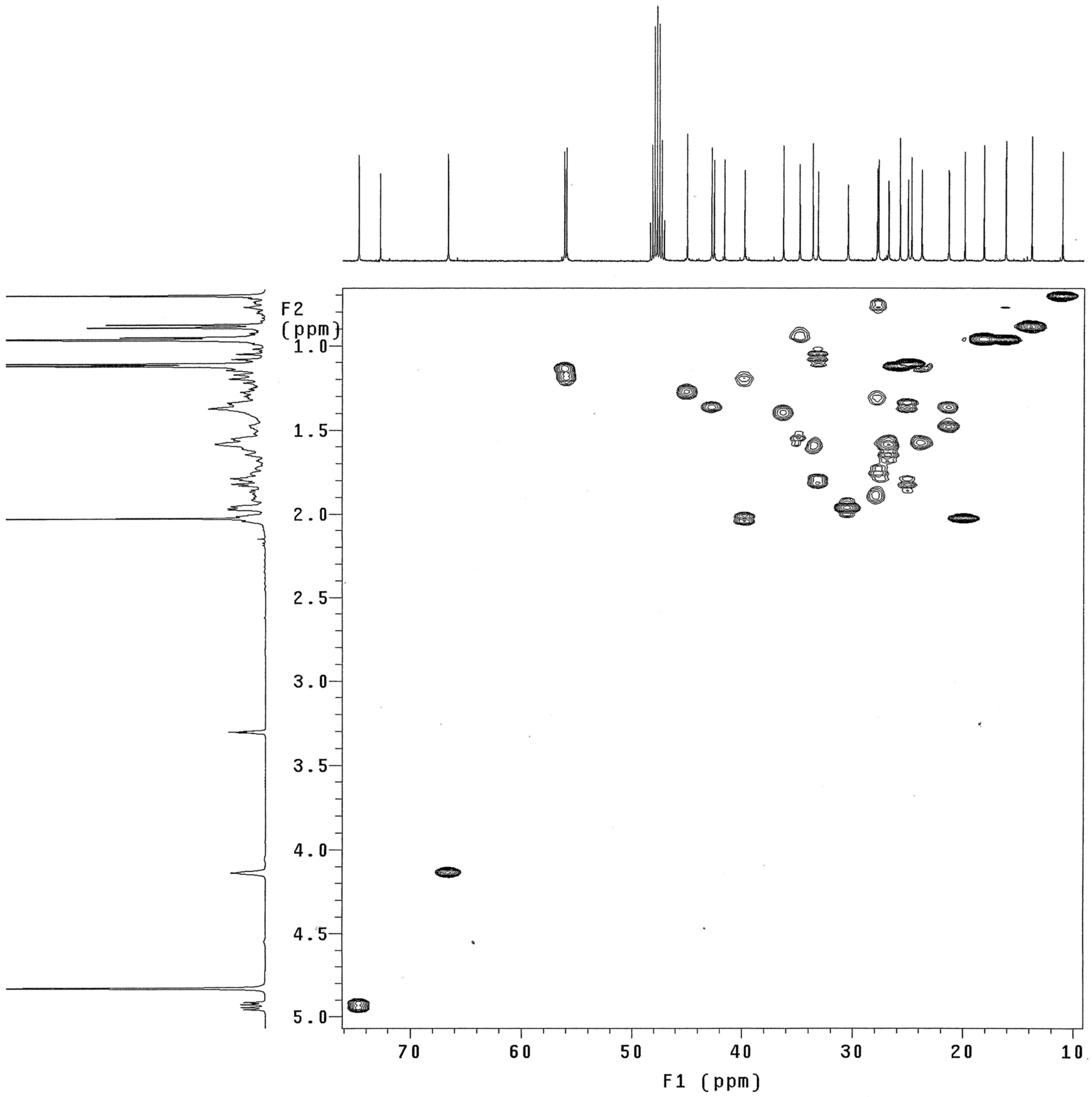

Figure S24. HSQC NMR spectrum of **3** in CD_3OD at 400 MHz.

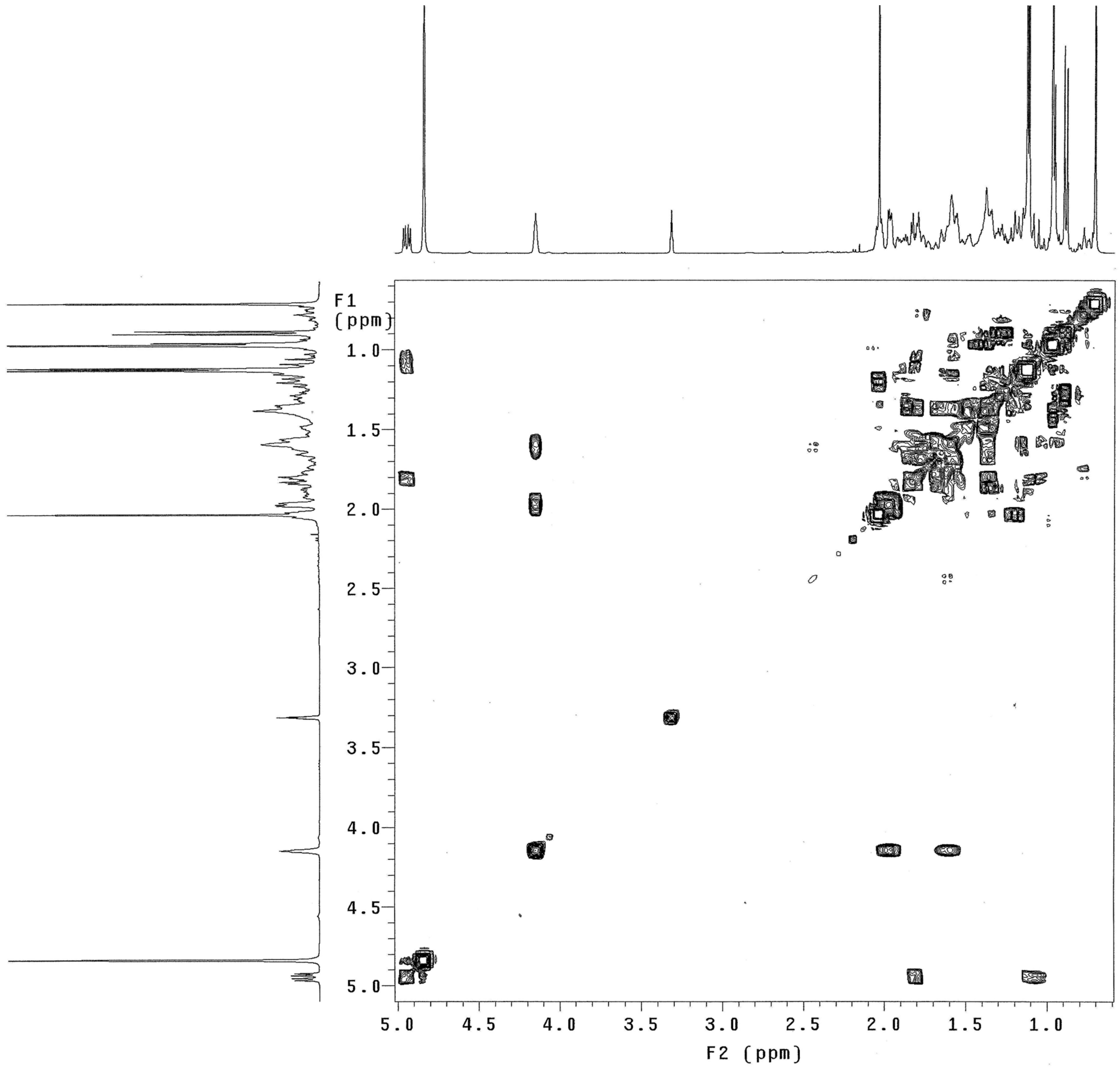

Figure S25. COSY NMR spectrum of **3** in CD_3OD at 400 MHz.

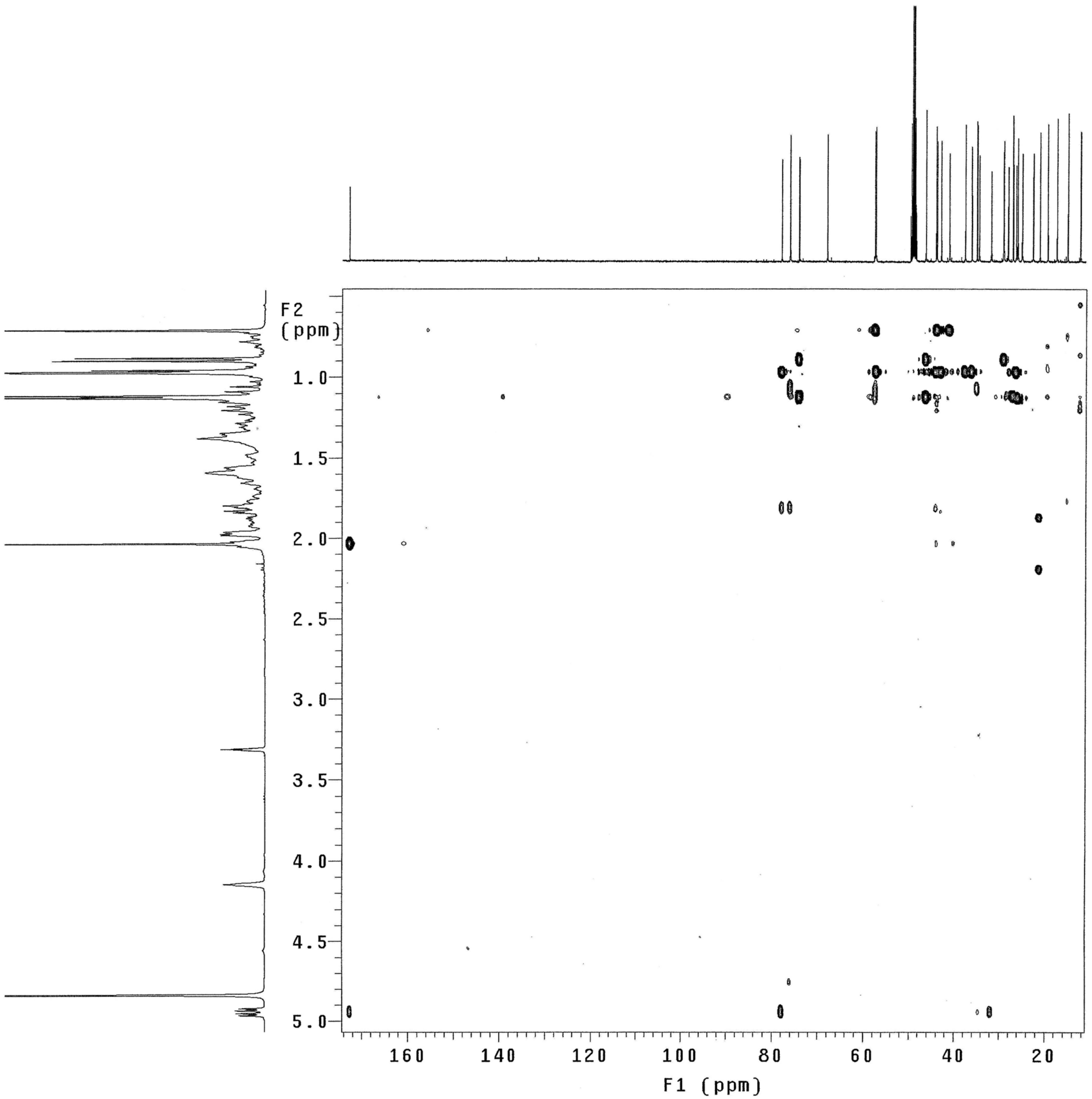

Figure S26. HMBC NMR spectrum of **3** in CD_3OD at 400 MHz.
